# Tumor-specific biochemical nanoconversion of self-assembled peptide-conjugated paclitaxel-docetaxel-based nanoparticles

**DOI:** 10.1186/s40580-025-00487-0

**Published:** 2025-04-26

**Authors:** Hansol Lim, Jae-Hyeon Lee, So-Hyeon Park, Jun-Hyuck Lee, Hyesu Jang, Seong-Bin Yang, Minho Seo, Seokwoo Lee, Jooho Park

**Affiliations:** 1 Department of Applied Life Science, BK21 Program, Konkuk University, Chungju, 27478 Republic of Korea; 2 College of Pharmacy, Chungnam National University, Daejeon, 34134 Republic of Korea; 3 Department of Biomedical Chemistry, College of Biomedical and Health Science, Konkuk University, Chungju, 27478 Republic of Korea

**Keywords:** Nanoconversion, Self-assembly, Cathepsin B, Docetaxel, Peptide conjugate, Nanomedicine

## Abstract

Docetaxel (DTX, **1**) and paclitaxel (PTX, **2**) are famous cytotoxic agents widely used in cancer therapy, however, their low specificity for tumor cells often results in severe systemic toxicity. Beyond conventional prodrug strategies, this study introduces a novel nanoconversion technology that chemically modifies DTX to form self-assembled nanoparticles (NPs), which subsequently convert into a paclitaxel-mimicking molecule (PTXm, **3**). Hydrophilic acetylated Phe-Arg-Arg-Phe peptide ((Ac)FRRF, **4**) and hydrophobic docetaxel were conjugated to prepare self-assembled (Ac)FRRF-DTX NPs. The selective cleavage of the Arg-Phe bond by cathepsin B, which is abundant in cancer cells, facilitated the nanoconversion of PTXm (**3**) from (Ac)FRRF-DTX NPs, demonstrating effective cytotoxic effects. Utilizing the cleavage site of peptide and specific sequences (ex. Arg-Arg-Phe), this approach does not simply act as a prodrug but allows the nanomaterial to transform into another cytotoxic biomolecule within tumors. (Ac)FRRF-DTX NPs exhibited remarkable physicochemical properties, superior anti-cancer efficacy, and low toxicity, showcasing an innovative conversion in peptide-conjugated nanomedicine. Unlike traditional prodrug chemistry, this tumor-specific nanoconversion process involves the biochemical transformation of DTX (**1**) into PTXm (**3**) via enzymatic action. Overall, this study provides an outstanding example of chemical drug molecular modification through the concept of nanoconversion.

**Supplementary Information:**

The online version contains supplementary material available at 10.1186/s40580-025-00487-0.

## Highlights

A novel nanoconversion strategy was developed in this study, enabling the transformation of docetaxel (DTX) into a paclitaxel mimic (PTXm) that is specifically activated by tumor-specific cathepsin B enzymes.

(Ac)FRRF-DTX, a conjugate of an acylated peptide and docetaxel (DTX), forms nanoparticles that self-assemble in saline solution.

Inactive (Ac)FRRF-DTX nanoparticles (NPs) are converted into active PTXm via cleavage by cathepsin B, which occurs specifically within the tumor microenvironment.

(Ac)FRRF-DTX, a peptide-drug conjugate, exhibits drug-like physicochemical properties, highlighting its potential as a novel drug delivery system.

**Supplementary Information:**

The online version contains supplementary material available at 10.1186/s40580-025-00487-0.

## Graphical Abstract

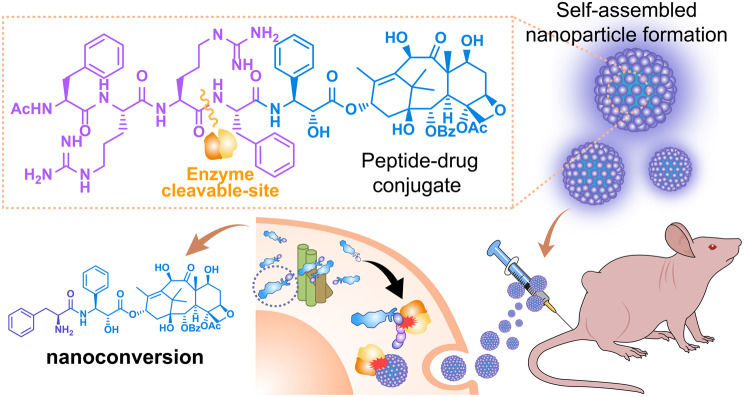

## Introduction

Biochemical molecular conversion is a reaction occasionally observed in nature and during drug metabolism. For example, prodrugs, which are inactive by themselves, undergo biochemical changes upon absorption into the body and are converted into different active molecules or therapeutic agents. Notably, various prodrugs utilizing drugs such as doxorubicin (DOX), docetaxel (DTX, **1**), and paclitaxel (PTX, **2**) have been developed in many previous studies [1–4]. These prodrug-type molecules are degraded or cleaved by specific enzymes in the body, to restore their original molecular structure and demonstrate therapeutic efficacy. Recently, in a similar concept, antibody-drug conjugates (ADCs) have also been included into this category, showing exceptional drug release efficacy [5, 6]. These primarily utilize a tumor-specific enzyme called cathepsin B, which is a lysosomal cysteine protease, to achieve peptide cleavage at the target tumor site [7, 8]. To improve cancer selectivity, extensive research has been conducted to adopt a prodrug strategy that involves enzymatic or chemical transformation into an active drug [9–11].

Selective activation of prodrugs can predominantly be achieved through various enzymes that are overexpressed in cancer cells [12, 13] and has been diversely utilized in combination with nanotechnology [14, 15]. Cathepsin B is a lysosomal cysteine protease that recognizes and cleaves various peptide sequences [16, 17], including Phe-Arg (FR), Arg-Arg (RR), Ala-Leu (AL), and Gly-Phe-Leu-Gly (GFLG), which are widely used in designing enzyme-cleavable nanoparticles [18–20]. Among them, dipeptide sequences (ex, RR) have been extensively utilized due to its high sensitivity and selectivity for cathepsin B.

In addition to other tumor-associated proteases, such as cathepsin D and matrix metalloproteinases (MMPs), also cleave specific sequences like Phe-Phe (FF) and Pro-Leu-Gly-Leu-Ala-Gly (PLGLAG), respectively [21, 22], supporting broader applications of enzyme-cleavable linkers in nanomedicine. Recent advances in pharmaceutical research have led to various applications of nanoparticles (NPs) owing to their capabilities for drug loading, controlled release, and specific targeting [23–25]. In particular, NPs have been extensively studied for cancer targeting due to their prolonged circulation half-life and the enhanced permeability and retention (EPR) effect [26–28]. However, the current focus remains on the delivery of active pharmaceuticals such as DOX, DTX (**1**), and PTX (**2**) through carriers. To the best of our knowledge, no studies on the biocompatible transformations of compounds within nanoparticles have been reported; Most nanomolecules utilize the concept of simple prodrug.

**Fig. 1:**
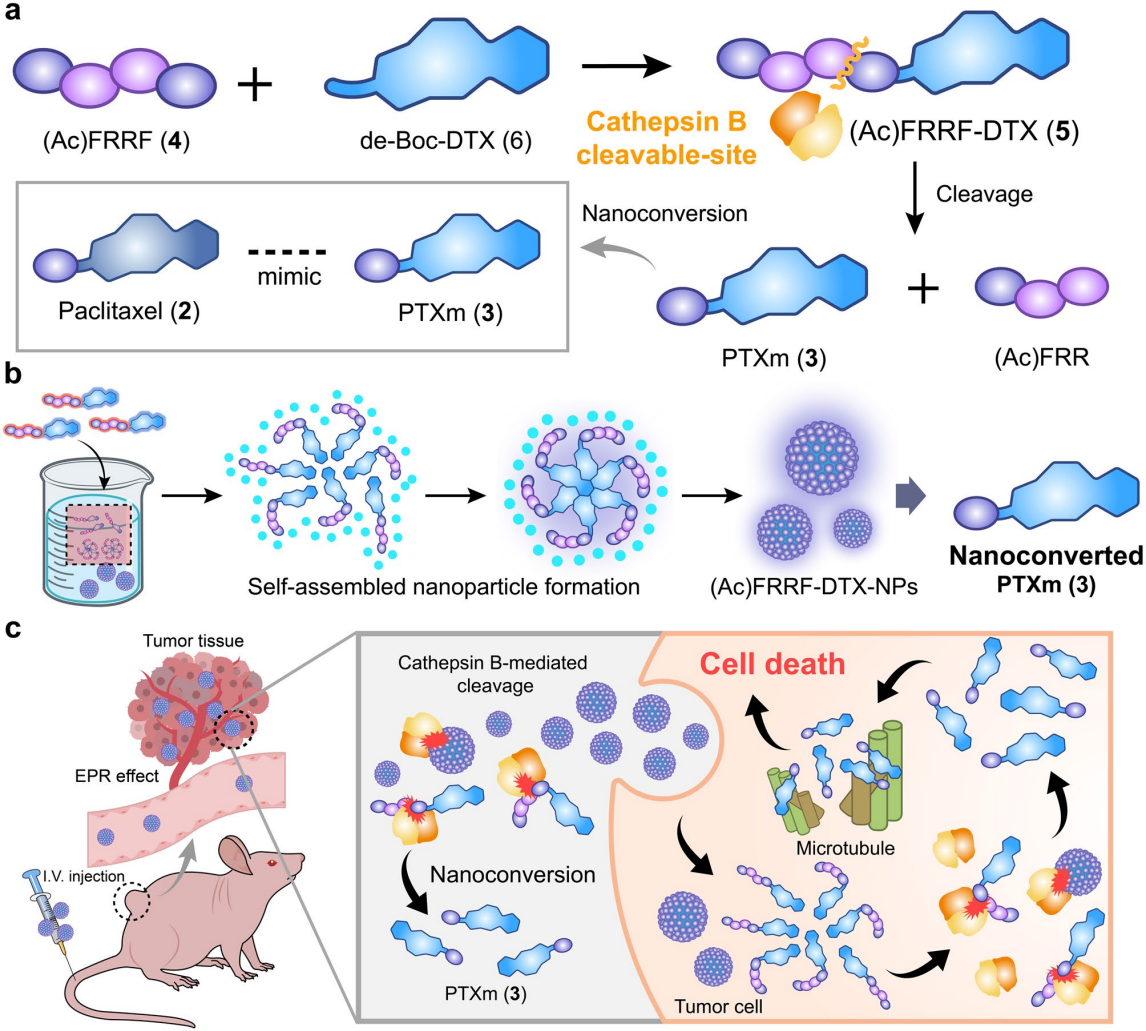
Schematic representation of (**a**) the molecular configuration and cathepsin B-cleavable design of (Ac)FRRF-DTX (5), enabling nanoconversion to paclitaxel-mimic PTXm (3). (**b**) the self-assembly of (Ac)FRRF-DTX (5) into nanoparticles in aqueous conditions. (**c**) Tumor-specific aggregation and inhibition of tumor growth by the conversion of (Ac)FRRF-DTX (5) to PTXm (3) through cathepsin B-mediated cleavage

In this study, we developed an innovative process for the nanoconversion of DTX (**1**) to a paclitaxel mimic (PTXm, **3**) via selective cleavage of a peptide (Phe-Arg-Arg, FRR) by cathepsin B, which is prevalent in tumor environments. First, DTX (**1**) was conjugated to the acylated peptide Phe-Arg-Arg-Phe ((Ac)FRRF, **4**) to yield the designated compound, (Ac)FRRF-DTX (**5**). Upon cathepsin B-mediated cleavage of the amide bond adjacent to the Arg (R) with retention of the terminal Phe (F) group, (Ac)FRRF-DTX (**5**) undergoes nonoconversion to PTXm (**3**), which exhibits bioactivity comparable to that of paclitaxel (Fig. 1a). Unlike conventional prodrugs, the initially loaded active compound, DTX (**1**), undergoes a sequence of in vivo transformations, leading to the intracellular formation of the novel compound PTXm (**3**) within the nano-assembly. Additionally, the incorporation of hydrophilic (Ac)FRRF (**4**) moiety into the hydrophobic DTX (**1**) backbone facilitated nanoparticle formation via self-assembly in saline (Fig. 1b). The resulting (Ac)FRRF-DTX nanoparticles ((Ac)FRRF-DTX NPs) can be administered intravenously and are expected to be selectively delivered to tumor tissues through the EPR effect. Subsequently a tumor-specific enzyme cathepsin B is anticipated to mediate the nanoconversion of (Ac)FRRF-DTX NPs into the active PTXm (**3**), which inhibits microtubule formation and thereby induces selective cytotoxicity (Fig. 1c). The design of a molecule that undergoes self-assembly and tumor-selective molecular transformation could serve as a novel example of innovative biochemical nanoconversion via biochemical reactions with low systemic toxicity. Moreover, the peptide-drug conjugate (PDC) manufactured in this study has remarkable physicochemical properties and has achieved successful self-assembly. This concept, which is delivered to the tumor with novel physicochemical properties of DTX and later converted to other cytotoxic agents, will be an example for future drug development.

## Materials and methods

### Materials

All chemicals were of reagent-grade and used as purchased. All reactions were performed under an inert atmosphere of dry nitrogen using distilled dry solvents. The reactions were monitored with TLC analysis using silica gel 60 F-254 thin layer plates. Compounds on the TLC plates were visualized under UV light and by spraying with either potassium permanganate or anisaldehyde solutions. Flash column chromatography was conducted on silica gel 60 (230–400 mesh). ^1^H and ^13^C NMR spectra were recorded on a Bruker Avance Neo 400 (400 MHz), Bruker Avance III 600 (600 MHz) or Bruker Avance III 600 and Bruker Avance III HD 800 MHz spectrometer equipped with a 5 mm triple resonance inverse (TCI) Cryoprobe at 298 K if not noted otherwise. Chemical shifts are reported in ppm (*δ*) units relative to the undeuterated solvent as a reference peak (D_2_O‒*d*_*2*_: 4.80 ppm/^1^H NMR, CD_3_OD‒*d*_*4*_: 3.30 ppm/^1^H NMR, 49.00 ppm/^13^C NMR). The following abbreviations are used to represent NMR peak multiplicities: s (singlet), d (doublet), t (triplet), m (multiplet), dd (doublet of doublets), dt (doublet of triplets), dq (doublet of quartets), td (triplet of doublets), and br (broad signal). High-resolution mass spectra (HRMS) were recorded using electrospray ionization (ESI) mass spectrometry on Sciex 1290 infinity II/TripleTOF 5600 plus.

Antibiotic antimycotic solution (100X), Bio TrackerTM 488 Green Microtubule Cytoskeleton Dye, Cathepsin B from bovine spleen, Dimethyl sulfoxide (DMSO), Dulbecco’s modified Eagle’s medium (DMEM), 1-ethyl-3-(3-dimethylaminopropyl) carbodiimide (EDCI), dichloromethane (DCM), ether, phosphate-buffered saline (PBS), rhodamine B isothiocyanate (RITC), 2-(4-amidi-nophenyl)-6-indolecarbamidine dihydrochloride (DAPI), Tetramethylsilane (TMS), Dimethyl sulfoxide-d6 (DMSO-d_6_) were purchased from Sigma-Aldrich (St. Louis, MO, USA). Paraformaldehyde solution 4% in PBS (PFA), PierceTM BCA Protein Assay Kit were obtained from Thermo Fisher Scientific (Waltham, MA, USA). EZ-Cytox kit was purchased from DoGenBio (Seoul, Republic of Korea). Fetal bovine serum (FBS), 0.5% Trypsin-EDTA (10X) were obtained from Gibico (Waltham, MA, USA). Neutral buffered formalin (10%) was purchased from HuBenTech (Damyang, Republic of Korea).

### Molecular dynamics (MD) simulations

All simulations were performed with GROMACS 2021_2 program. (Ac)FRRF-DTX (**5**) were described by CHARM36m force field and parameters generated by CHARMM-GUI web server. The tip3 was used for solvent model. All systems were neutralized by the addition chloride ions (Cl^−^) and sodium ion (Na^+^). The time-step was set to 100 ns, and a cutoff of 1.4 nm was used for short-range van der Waals and electrostatics. Long range electrostatics were performed with the Particle-Mesh-Ewald method using a Fourier spacing of 0.24 nm and fourth order interpolation. Bonds were constrained using the LINCS algorithm and rigid water Temperature coupling was performed with the v-rescale thermostat while pressure coupling was performed with the Berendsen barostat during equilibration and the Parrinello-Rahman barostat during sampling. All simulations were run at 300 K and 1 bar. The trajectories generated from the (Ac)FRRF-DTX nanoparticle formation experiment executed in the GROMACS 2021_2 program was saved as PDB files using PyMOL software (Version 2.5.0; Schrödinger, LLC). Subsequently, all trajectories inputted in PDB format were analyzed using the Analyze Trajectory module in Discovery Studio 2022 (v22.1.0.) software (BIOVIA; California, USA). To measure the number of hydrogen bonds formed during the formation of nanoparticles by each molecule, a monitor for H bonds was applied. Additionally, monitors were applied in a similar manner to measure the number of electrostatic bonds and hydrophobic interactions formed by the hydrophilic and hydrophobic regions of each molecule. To assess the similarity between PTX (2) and PTXm (3) cleaved by cathepsin B, quantum mechanics were conducted. The calculations were performed using DMol^3^ program in Discovery Studio 2022 software for HOMO and LUMO energy. The Perdew–Wang (PWC) local density approximation (LDA) exchange–correlation functional was applied, and a double numerical plus polarization (DNP) basis set was employed.

### Characterization of (Ac)FRRF-DTX

To verify the synthesis and purification of (Ac)FRRF-DTX (5), it was dissolved in distilled water at a concentration of 1 mg/mL. The compound was then analyzed using liquid chromatography-mass spectrometry (LC-MS). The synthesis and purification of (Ac)FRRF-DTX (5) were confirmed using reverse-phase high-performance liquid chromatography (RP-HPLC), which included the reaction components (Ac)FRRF (4), de-Boc-DTX (6), PTX (2) and DTX (1). During the measurement, the detectable mass range was set between 500 and 1500, and the analysis was performed under positive ion mode with a parameter setting of 3000. The particle size of (Ac)FRRF-DTX (5) (0.25 mg, 0.184 µmol) was measured in DW at a concentration of 0.25 mg/mL using dynamic light scattering (DLS; Zeta Sizer Nano, Malvern Instruments, Worcestershire, UK). (Ac)FRRF-DTX (5) nanoparticles were prepared by dissolving 0.5 mg of (Ac)FRRF-DTX (5) in 2 mL of filtered DW through a simple process. For measurements, the solution was sonicated for 5 min at RT using JAC Ultrasonic 3010 (KODO, Republic of Korea). The particle stability of (Ac)FRRF-DTX (5) (0.1 mg, 0.0737 µmol) was assessed in DW (0.1 mg/mL) using DLS. After gentle dissolution, (Ac)FRRF-DTX (5) nanoparticles were evaluated at various time points (0, 1, 3, 6, 12, 24, 48 and 72 h) (*n* = 3). The morphologies of (Ac) FRRF (4) (0.2 mg, 0.3 µmol), de-Boc-DTX (6) (0.2 mg, 0.283 µmol), and (Ac)FRRF-DTX (5) (1 mg, 0.737 µmol) were observed using transmission electron microscopy (TEM; EVO MA 10, Carl Zeiss, Germany) and field emission scanning electron microscopy (FE-SEM; MERLI; Carl Zeiss, Germany).

### 2.4. Solubility assessment of (Ac)FRRF-DTX

To assess the solubility of (Ac)FRRF-DTX, a solubility evaluation was conducted along with DTX and PTX. Solubility evaluation images were captured for solutions where DTX, PTX, and (Ac)FRRF-DTX were dissolved at a specified concentration (0.3 mg/mL) in a mixture of 3% DMSO and DW. The images aimed to observe how well these compounds dissolved in the solution. Additionally, an experiment was carried out to precisely determine the aqueous solubility values of (Ac)FRRF-DTX nanoparticles. The solvent conditions for both the image evaluation and the experiment involved a solution where 3% DMSO was mixed with DW. These evaluations provide visual observations of the solubility of DTX, PTX, and (Ac)FRRF-DTX in the specified solvent conditions. Moreover, complementary experiments were performed to obtain accurate quantitative solubility values for (Ac)FRRF-DTX NPs.

### Cathepsin B cleavage assay

The cathepsin B-specific cleavage of (Ac)FRRF-DTX was analyzed using reverse-phase high-performance liquid chromatography (RP-HPLC), and the molecular weight of PTXm was determined by liquid chromatography-mass spectrometry (LC-MS). In brief, nanoparticles of 50 µM (Ac)FRRF-DTX (0.0678 mg, 0.05 µmol) were cultured with cathepsin B (50 µg/mL) at 37℃ in 25 mM 2-(N-morpholino)-ethanesulfonic acid (MES) buffer at various time point (0, 1, 3, 6, 12, 24, 48 and 72 h). To confirm the cleavage status of (Ac)FRRF-DTX in an environment lacking cathepsin B, a control group without cathepsin B treatment was processed using the same method. The cleavage profile of (Ac)FRRF-DTX NPs was analyzed at 245 nm using reversed-phase high-performance liquid chromatography (RP-HPLC) under solvent gradient conditions (from 90:10 H_2_O/acetonitrile to 10:90 H_2_O/acetonitrile over 40 min). Furthermore, the HPLC data of PTXm was observed at a concentration of 0.3 mg/mL dissolved in MES buffer 1X, following the same method as (Ac)FRRF-DTX for cathepsin B cleavage assay. Molecular weight analysis of the PTXm was performed by liquid chromatography-mass spectrometry (LC-MS) using solvent gradient conditions (from 90:10 H_2_O/acetonitrile to 10:90 H_2_O/acetonitrile over 20 min) in scan mode at 2000 parameter values. The conditions were confirmed with a minimum and maximum molecular weight of 800 to 1200, respectively.

### Cathepsin B activity

CT26.wt, Hep G2, A549, and HDFa cells were all cultured in high-glucose DMEM medium containing 10% FBS, and the cell count for each was adjusted to 1.0 × 10^6^ cells. Subsequently, the cells were lysed using a cathepsin B activity assay kit (Sigma-Aldrich, Milwaukee, WI, USA). The cell lysates were centrifuged at 13,000 rpm, for 5 min at 4℃, and the supernatant was collected and kept on ice. For the cathepsin B activity assay, 48 µL of the collected protein from the supernatant was mixed with 50 µL of the reaction buffer from the cathepsin B activity assay kit and 2 µL of Ac-RR-AFC (1000 µM). The mixture was then dispensed into a 96-well black plate (non-treated) with 100 µL per well. The plate was maintained at 37℃ for 2 h, and the fluorescence of each well was measured using a SpectraMax M2 microplate reader (λ_Ex_ = 400 nm / λ_Em_ = 505 nm wavelength).

### In vitro cytotoxicity assay

The cytotoxicity of (Ac)FRRF, de-Boc-DTX, (Ac)FRRF-DTX NPs, PTXm and PTX were evaluated using the EZ-Cytox assay on Hep G2 cell. Hep G2 was cultured in a 96-well plate at concentrations of 5.0 × 10^3^ cells/well and 1.0 × 10^4^ cells/well, respectively, under conditions of 37℃ and 5% CO_2_ for 1 h. Subsequently, cells were cultured for 48 h with concentrations ranging from 0.1 to 500 µM of each drug. Cell viability (*n* = 5 − 6) was measured at 450 and 600 nm absorbance using the EZ-Cytox cell viability assay kit (Daeil Lab Service, Republic of Korea) and a microplate reader (SPECTROstar Nano spectrophotometer, BMG Labtech, Germany). Cell viability was calculated by comparing the absorbance values of the measured samples with those of the control group. Additionally, to more accurately confirm the cytotoxicity of DTX and PTX with low solubility in aqueous media for Hep G2 cell, both of drug were dissolved in a 3% DMSO solution and cultured for 48 h at concentrations ranging from 0.1 to 500 µM. Cell viability was determined using the same method.

### In vitro cellular uptake assay

To investigate the cellular uptake of (Ac)FRRF-DTX NPs in comparison to PTX in Hep G2 cells, both materials were fluorescently labeled with rhodamine B isothiocyanate (RITC). Hep G2 cells were cultured in high-glucose DMEM medium and maintained in a sample chamber at 37℃ with 5% CO_2_ and proper humidity. The cells were pre-incubated overnight at a density of 5 × 10^4^ in 35 mm fluorescent cell culture dishes (SPL Life Science, Republic of Korea). Subsequently, to observe the intracellular uptake of nanoparticles, each material was administered at a concentration of 10 µM at different time points (1, 2, 4 and 8 h) and then fixed. The nuclei were stained with DAPI, and images were captured using the ECLIPSE Ti2 series (Nikon, Japan) with an RITC filter. Additionally, the fluorescence intensity of RITC was quantified using ImageJ (U.S. National Institutes of Health) (*n* = 3). Additionally, the precise intracellular localization of (Ac)FRRF-DTX NPs was confirmed using a confocal microscope. Hep G2 cells were cultured overnight at a density of 1.0 × 10^4^ in 35 mm black confocal dishes (SPL Life Science, Republic of Korea). Subsequently, the cells were treated with RITC-labeled (Ac)FRRF-DTX NPs at a concentration of 100 µM for 24 h. After washing, the cells were fixed with 4% paraformaldehyde (4% PFA) and stained with phalloidin and DAPI to visualize the cytoskeleton and nuclei in green and blue fluorescence, respectively. Images were then acquired using a confocal laser scanning microscope (CLSM), LSM980 (Carl Zeiss, Germany), at the wavelengths corresponding to FITC, DAPI and RITC.

### In vitro nucleic green evaluation

To assess the cell death of Hep G2 cells induced by PTX, PTXm, and (Ac)FRRF-DTX NPs, the cells were evaluated using nucleic green reagent and the results were observed through imaging. Hep G2 cells were cultured in high-glucose DMEM medium, maintained in a sample chamber with appropriate humidity, and kept at 37℃ with 5% CO_2_. The cells were cultured in 35 mm fluorescent cell culture dishes (SPL Life Science, Republic of Korea) at a concentration of 4 × 10^4^ for 3 h. Subsequently, DTX, PTX, and (Ac)FRRF-DTX NPs were all treated at a concentration of 100 µM (in DMSO 2%) for 24 h. After sample treatment, cells were washed twice with DPBS solution, fixed with 4% PFA. The fixed cells were further treated with SYTOXTM Green nucleic acid stain (Invitrogen, Waltham, USA, 1 µM) at RT for 15 min in a light-protected clean bench. Following this, the cells were washed three times with cold DPBS, and images were captured using ECLIPSE Ti2 Series (Nikon, Japan) through a FITC filter while keeping the samples in DPBS.

### In vitro microtubule staining

To assess the cell death induced by PTX, PTXm, and (Ac)FRRF-DTX NPs in Hep G2 cells, an evaluation was conducted using the nucleic green reagent, and the results were visualized through imaging. Hep G2 cells cultured in high-glucose DMEM medium, maintain in a sample chamber with appropriate humidity, and kept at 37℃ with 5% CO_2_. The cells were cultured in 35 mm fluorescent cell culture dishes (SPL Life Science, Republic of Korea) at a concentration of 4 × 10^4^ for 3 h. Subsequently, DTX, PTX, and (Ac)FRRF-DTX NPs were all treated at a concentration of 100 µM (in DMSO 2%) for 24 h. After sample treatment, cells were washed five times with DPBS solution, and in a light-protected environment, the medium was replaced with a mixture of microtubule reagent (Bio Tracker™ 488 Green Microtubule Cytoskeleton Dye, Sigma-Aldrich, MO, USA) in fresh medium at RT for 30 min. Following this, the medium was replaced with new DMEM containing 10% FBS, and images were captured using ECLIPSE Ti2 Series (Nikon, Japan) with a FITC filter and quantified using ImageJ (U.S. National Institutes of Health).

### Biodistribution of (Ac)FRRF-DTX

To assess the in vivo distribution of (Ac)FRRF-DTX NPs, 6-week-old male BALB/c nude mice (Orient Bio, Seongnam, Republic of Korea) were utilized. To confirm the distribution of the substance in tumor tissue, Hep G2 were subcutaneously implanted in the mice, and the experiment was conducted when the tumor size reached 100 mm^3^. For distribution confirmation, fluorescently labeled RITC-(Ac)FRRF-DTX (20 mg/kg) was administered via intravenous injection, with each substance dissolved in 200 µL of saline. The fluorescence intensity of RITC-(Ac)FRRF-DTX was measured and quantified using a precision fluorescence analyzer (FOBI, CELLGENTEK, Republic of Korea) at various time points after intravenous injection (0, 0.5, 1, 3, 6, 12, 24 and 48 h). Anesthesia for fluorescence intensity measurement involved intravenous injection of 2,2,2-tribromoethanol (25 g), induced by 60-fold diluted tetra amyl alcohol (15.5 mL) in purified saline. Additionally, to compare and analyze the in vivo distribution of (Ac)FRRF-DTX NPs more accurately with de-Boc-DTX, 6-week-old male BALB/c nude mice (Orient Bio, Seongnam, Republic of Korea) were used. RITC-de-Boc-DTX and RITC-(Ac)FRRF-DTX NPs (20 mg/kg) were administered via the mouse tail vein, and major organs (liver, kidney, lung, heart, spleen) and tumor tissues were harvested at various time points after injection (6, 12, 24 and 48 h). Fluorescence intensity was measured and quantified using a precision fluorescence analyzer. All mice used in the experiment were fasted for 18 h before administration, and after 48 h of substance administration, they were euthanized.

### The retention level in the bloodstream experiment

The retention level in the bloodstream of de-Boc-DTX and (Ac)FRRF-DTX NPs with RITC fluorescence labeling in the serum of male Sprague-Dawley rats was investigated. RITC-de-Boc-DTX and RITC-(Ac)FRRF-DTX were administered via the tail vein at a dose of 0.1 mg/kg each. For this, a saline solution with a concentration of 200 µL per rat (200 g) was used. Whole blood samples were collected from the jugular vein of rats at specified time points (0.1, 0.5, 1, 3, 6, 12 and 24 h) post intravenous injection. The blood samples were coagulated RT for 30 min and then centrifuged at 4500 g for 15 min at 4℃ to separate serum. The collected serum was mixed with DMSO to make a final solution with 20% DMSO and was dispensed into 96-well black plate (non-treated) with 100 µL per well. Subsequently, the fluorescence of each well was measured using a SpectraMax M2 microplate reader (λ_Ex_ = 555 nm/λ_Em_ = 595 nm wavelength).

### In vivo antitumor test

All animal experiments were conducted in accordance with the standard regulations of the Konkuk University Animal Experimentation Ethics Committee (Ref: no. KU220748-1). Hep G2 cells at a concentration of 1.0 × 10^7^ cells/100 µL were subcutaneously inoculated into the dorsal region at 6-week-old male BALB/c nude mice (Orient Bio, Seongnam, Republic of Korea). After 9 weeks, when the tumor volume reached approximately 50 − 100 mm^3^ (calculated as the maximum diameter × minimum diameter^2^ × 0.52), the mice were divided into four groups. Mice with tumors were intravenously administered saline, de-Boc-DTX (5 mg/kg), PTX (5 mg/kg), or (Ac)FRRF-DTX NPs (10 mg/kg; based on 5 mg/kg of de-Boc-DTX) at 3 days intervals for a total of 17 days. The body weight and tumor volume of the treated mice were measured every other day using a digital caliper (ASIMETO, Mooresville, NC, USA). After 17 days of treatment, tumors were fixed with diluted 10% neutral pH formalin (HuBen Tech, Republic of Korea). Tumor tissues were stained with TUNEL, Ki-67, and H&E. Sections of tumor tissues stained with Ki-67 and H&E were observed using the OS-33DPM multimedia video microscope (OSUN HITECH, Republic of Korea). Additionally, tumor tissues stained with TUNEL were sliced for histological observation using an ECLIPSE Ti2 inverted microscope (Nikon, Japan), and FITC fluorescence intensity was quantified using ImageJ (U.S. National Institutes of Health) (*n* = 3).

### In vivo blood toxicity assessment

Hematological analysis of red blood cell (RBC) and white blood cell (WBC) parameters in mice treated with (Ac)FRRF-DTX was performed through clinical pathological examinations. Normal saline (control group), de-Boc-DTX (1.5 mg/kg), PTX (1.5 mg/kg), and (Ac)FRRF-DTX NPs (3 mg/kg) were dissolved in 200 µL of normal saline and administered daily via tail vein. After 7 days, blood was drawn from the mice’s heart and collected in tubes containing EDTA to prevent coagulation. Subsequently, whole blood samples were stored for analysis. Level of RBC-related parameters (RBC, HGB, HCT, MCV, MHC, MCHC) and WBC-related parameters (WBC, PLT, NEU, LYM, MONO, EOS) were measured using a hematology analyzer (Sysmex XN series, Sysmex, Japan) (*n* = 5).

### In vivo organ toxicity assessment

Clinical pathological analysis was conducted to assess the hematological parameters related to the liver and kidney in mice treated with (Ac)FRRF-DTX NPs. Following the completion of the antitumor experiment, whole blood was collected from the mice’s hearts and allowed to clot at RT for 30 min. The clotted blood was then centrifuged at 4℃ for 15 min at 4500 g to separate serum. The obtained serum was subjected to biochemical analysis using a clinical analyzer (7180 Clinical Analyzer, HITACHI, Japan) to measure levels of aspartate aminotransferase (AST), alkaline phosphatase (ALP), lactate dehydrogenase (LDH), lactate (LAC), total protein (TP), albumin (Alb), albumin/globulin ratio (A/G), blood urea nitrogen (BUN), and creatinine (Crea).

### Statistical analysis

Statistical analyses were carried out using GraphPad Prism (version 9.0, GraphPad Software Inc.). All values are presented as mean ± standard deviation (S.D.), and error bars represent the S.D. derived from independently conducted experiments. To determine statistical differences among groups, one-way ANOVA followed by appropriate post hoc comparisons was employed. Significance levels were defined as *p < 0.05, **p < 0.01, and ***p < 0.001, respectively.

## Results and discussion

### Design and computer modeling for nanoconversion

**Fig. 2:**
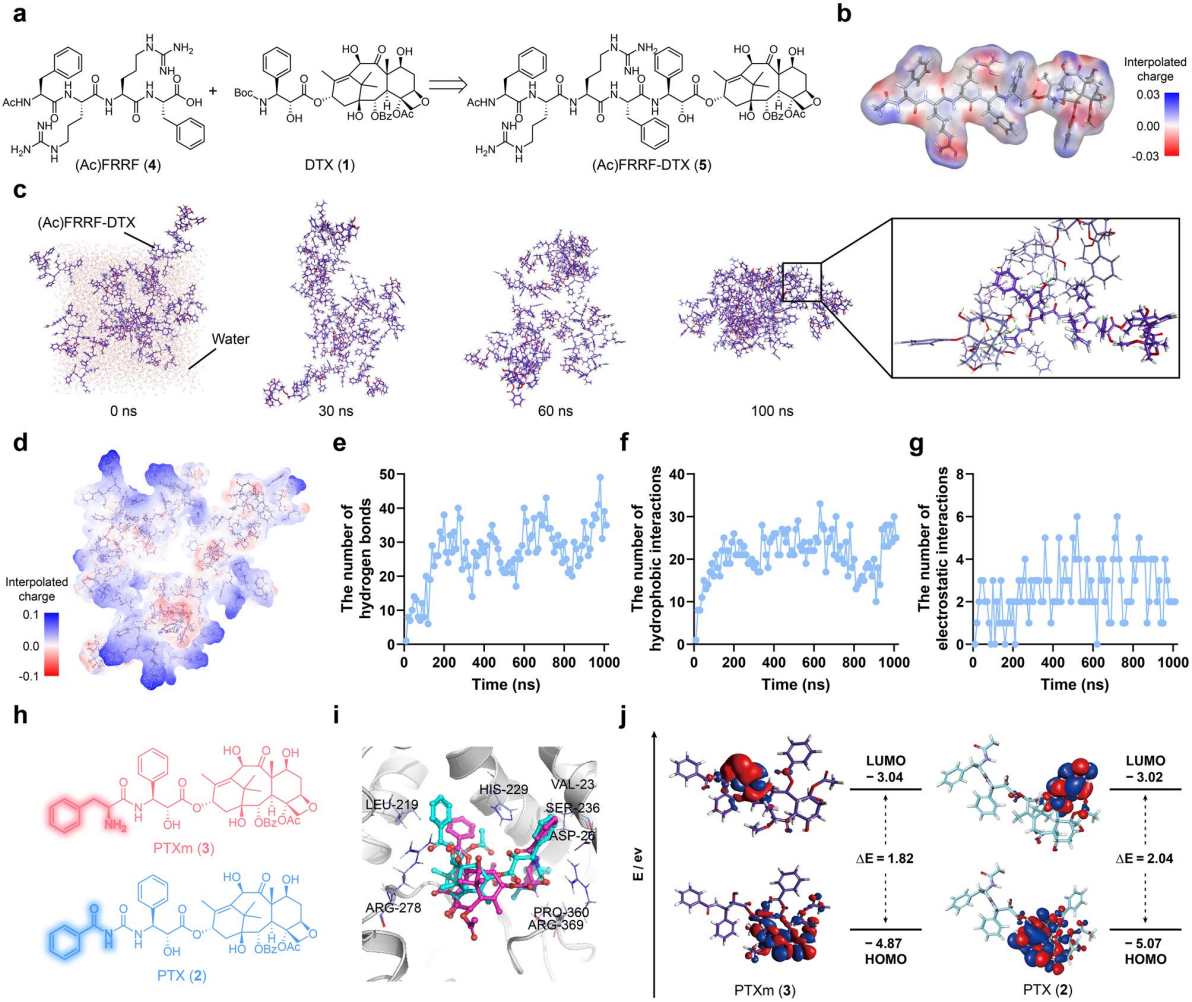
In silico studies of (Ac)FRRF-DTX (5) and PTXm (3). (**a**) Synthetic plan of (Ac)FRRF-DTX (5). (**b**) Interpolated charge surface of the amphiphilic (Ac)FRRF-DTX (5). (**c**) Molecular dynamics simulation of self-assembled (Ac)FRRF-DTX NPs in an explicit solvent model. (**d**) Overall interpolated charge of (Ac)FRRF-DTX NPs. The number of hydrogen bonds (**e**), hydrophobic interactions (**f**), and electrostatic interactions (**g**) of (Ac)FRRF-DTX NPs based on molecular dynamics (MD) simulation. (**h**) Chemical structure of PTX (2) and PTXm (3). (**i**) Comparative docking affinity analysis of microtubule interactions between PTX (2) and PTXm (3) through Assisted Molecular Docking (AMdock). (**j**) DFT calculation with Discovery Studio Discovery Studio using LDA-PWC method and DNP basis set in PTX (2) and PTXm (3)

Our designed (Ac)FRRF-DTX (5), based on hydrophilic (Ac)FRRF (4) and hydrophobic DTX (1) as shown in (Fig. 2a). Before the synthesis, to gain insight into the potential self-assembly properties of (Ac)FRRF-DTX (5) for nanoconversion via in silico study as like our previous study [29]. First, the amphiphilic properties of (Ac)FRRF-DTX (5) were validated through an electrostatic potential map (Fig. 2b). Next, to uncover the self-assembly of (Ac)FRRF-DTX (5) the molecular dynamics (MD) simulations were carried out and observed snapshots of the simulation system at different time points (0, 30, 60, and 100 ns). (Ac)FRRF-DTX (5), along with sodium and chloride ions, were distributed in a cubic water box and eventually aggregated into a single cluster within the solution system, forming intermolecular interaction within 100 ns (Fig. 2c). After cluster formation, the hydrophobic DTX (1) moiety predominantly occupied the interior (neutral in white), whereas the hydrophilic (Ac)FRRF (4) moiety was preferentially distributed along the exterior (negatively charged in red and positively charged in blue, Fig. 2d). In detail, as time elapsed, the hydrogen bonds, hydrophobic interactions, and electrostatic interactions progressively increased, indicating that our self-assembly is spontaneous and becoming more stabilized (Fig. 2e − g).

Additionally, the biological activity of PTXm (3), generated via nanoconversion, was modeled to assess its similarity to PTX (2) (Fig. 2h). Using the microtubule crystal structure (PDB: 5M5O), molecular docking studies were performed on the interaction of PTXm (3) or PTX (2). In our docking models, PTXm (3) exhibited a binding conformation and affinity similar to PTX (2) (–8.0 vs. − 8.9 kcal/mol), maintaining crucial interactions with microtubule residue LEU219, HIS229, SER239, ARG278, PRO360, and ARG369 (Fig. 2i and Fig. S1). These findings suggest that PTXm (3) is likely to exhibit comparable biological activity, as it forms similar binding interactions with microtubules, the primary targets of taxol-based compounds. This similarity can be attributed not only to the geometric resemblance between PTXm (3) and PTX (2) but also to their electronic similarity, as evidenced by comparable HOMO-LUMO energy gaps (1.82 vs. 2.04 eV, Fig. 2j and Table. S1 − S2). Based on in silico results, (Ac)FRRF-DTX (5) was determined to form nanoparticles through self-assembly, and the resulting PTXm (3), produced via selective nanoconversion through targeted cleavage, was anticipated to exhibit bioactivity similar to that of PTX (2).

### Synthesis and evaluation of (Ac)FRRF-DTX NPs

**Fig. 3:**
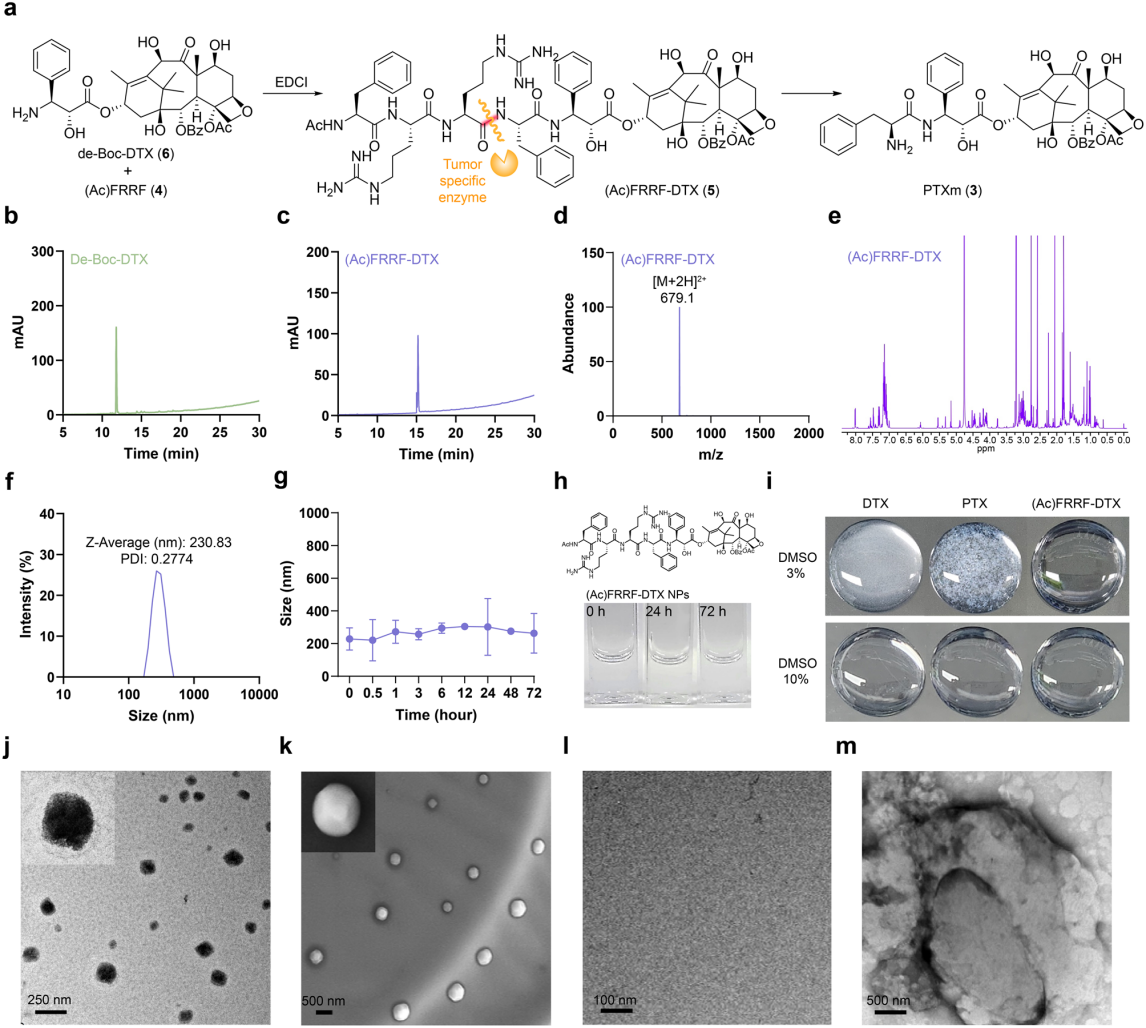
Preparation and characterization of (Ac)FRRF-DTX and (Ac)FRRF-DTX NPs. (**a**) Synthesis of (Ac)FRRF-DTX and nanoconversion for PTXm via cathepsin B. High-performance liquid chromatography (HPLC) analysis of de-Boc-DTX (**b**) and (Ac)FRRF-DTX (**c**), LC-MS analysis of (Ac)FRRF-DTX (**d**), and NMR analysis of (Ac)FRRF-DTX (**e**). (**f**) Size distribution of (Ac)FRRF-DTX NPs. (**g**) Size stability evaluation of (Ac)FRRF-DTX NPs over time (*n* = 3). (**h**) Time-dependent dissolution images. (**i**) Solubility images of DTX, PTX, and (Ac)FRRF-DTX in 3% DMSO and 10% DMSO solvents. Morphological images of (Ac)FRRF-DTX NPs by TEM (**j**) and SEM (**k**). TEM image of (Ac) FRRF (**l**) and de-Boc-DTX (**m**)

Our synthesis began with the preparation of a known amine, de-Boc-DTX (6), by deprotecting the *N*-Boc group of commercially available DTX (1) [30]. The (Ac)FRRF peptide (4) possessing a cathepsin B selective cleavage moiety (FRR) was prepared by stepwise solid-phase peptide synthesis. Subsequently, to synthesize (Ac)FRRF-DTX (5), which is capable of nanoconversion, de-Boc-DTX (6) was coupled with (Ac)FRRF (4) using EDCI as a coupling agent (Fig. 3a). Each compound was confirmed by high-performance liquid chromatography (HPLC), and the purity of (Ac)FRRF-DTX (5) was found to be approximately 98% (Fig. 3b and c and Fig. S2). In addition, the molecular weight of (Ac)FRRF-DTX (5) was confirmed by liquid chromatography mass spectrometry (LC-MS), and the formation of (Ac)FRRF-DTX (5) was confirmed by the downfield α-proton of the free amine and high-resolution mass spectrometry (HRMS) (Fig. 3d and e).

(Ac)FRRF-DTX (5) nanoparticles ((Ac)FRRF-DTX NPs) were prepared using a one-step nanoprecipitation method according to our previous reports [31–33]. As anticipated in previous in silico studies, amphiphilic (Ac)FRRF-DTX (5) could spontaneously self-assemble into uniform nanoparticles after dispersion in water. The particle size of (Ac)FRRF-DTX NPs was 230.83 ± 46.20 nm (PDI = 0.2774) with a relatively homogenous size distribution (Fig. 3f) and zeta potential of (Ac)FRRF-DTX NPs was 21.91 ± 4.70 mV. Although the observed size of the nanoparticles (~ 230 nm) may seem larger than what is typically expected for micelles or lipid nanoparticles (LNPs), nanoparticles with an average size of around 230 nm have been widely reported to be effective in drug delivery [34–36]. Furthermore, the mixed solution maintained a clear and well dissolved state for 72 h, with no substantial alteration in particle size was observed during this period (Fig. 3g and h). Additionally, the differences in solubility between DTX (1), PTX (2), and (Ac)FRRF-DTX (5) were assessed at a concentration of 0.3 mg/mL in water with either 3% or 10% DMSO. In 10% DMSO, all three solutions appeared clear, whereas in 3% DMSO, DTX (1) and PTX (2) became opaque or precipitated, while (Ac)FRRF-DTX (5) remained fully dissolved at the concentration 0.3 mg/mL (Fig. 3i). Unlike hydrophobic DTX and PTX, (Ac)FRRF-DTX (5), conjugated to the hydrophilic (Ac)FRRF peptide residue, exhibits amphiphilic properties that facilitate its dissolution in aqueous media. Notably, (Ac)FRRF-DTX (5) exhibited a high solubility of 1.3 mg/20 µL in water without DMSO. The morphology of (Ac)FRRF-DTX NPs was characterized as a spherical vesicle, a characterization supported by transmission electron microscopy (TEM) and scanning electron microscopy (SEM) studies (Fig. 3j and k). In the control group, composed solely of either (Ac)FRRF (4) or de-Boc-DTX (6), absence of nanoparticle formation (Fig. 3l and m) emphasized the pivotal importance of chemical bonding between these two compounds.

### Nanoconversion and cytotoxicity assessment of (Ac)FRRF-DTX NPs by cathepsin B

**Fig. 4:**
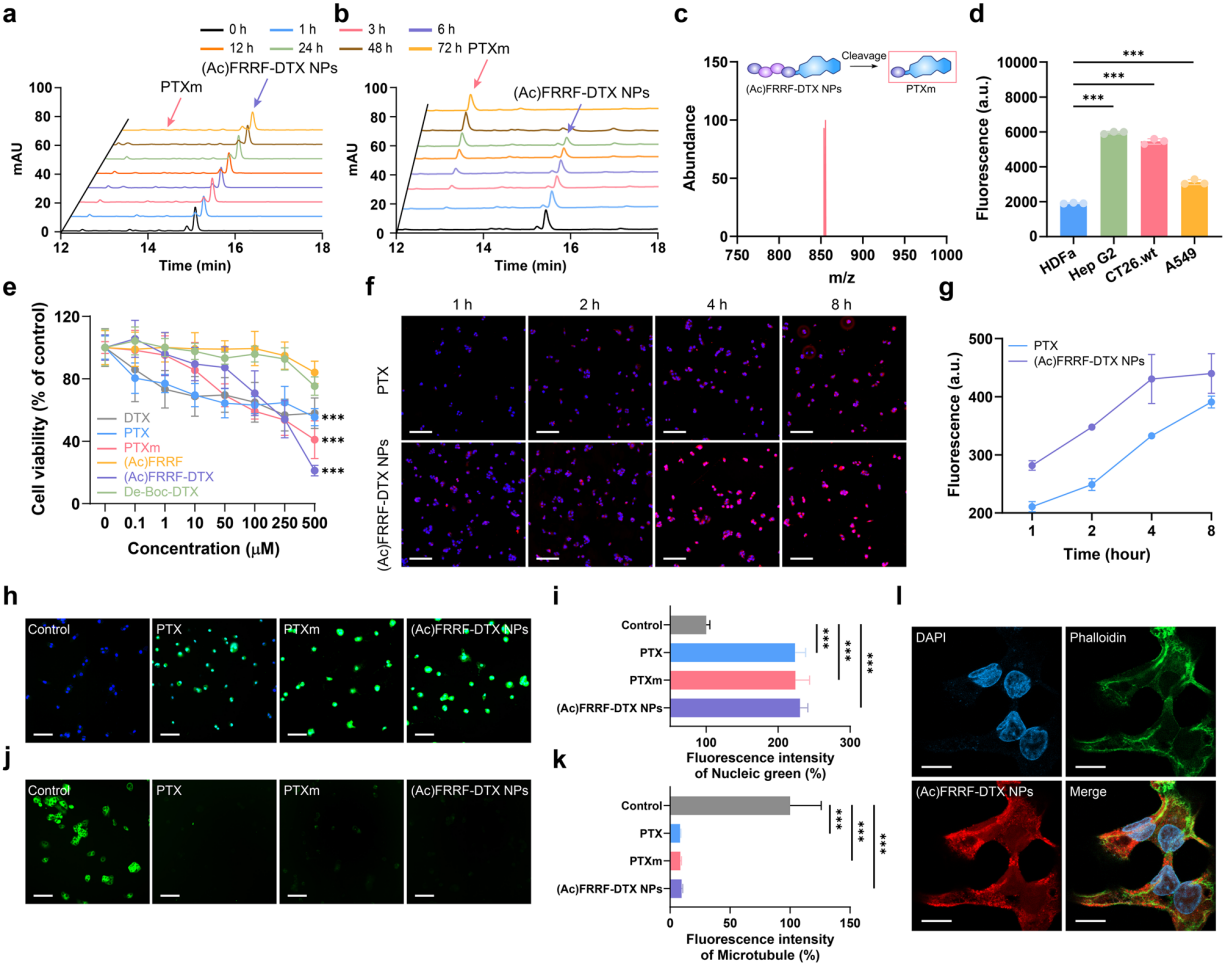
In vitro, cathepsin B cleavage, cellular toxicity and fluorescence imaging of (Ac)FRRF-DTX NPs. HPLC analysis of (Ac)FRRF-DTX NPs incubated without (**a**) or with (**b**) enzyme reaction buffer containing cathepsin B for different time points (0–72 h). (**c**) LC-MS analysis of PTXm during the cathepsin B cleavage experiment. (**d**) The assessment of cathepsin B activity levels in HDFa, Hep G2, CT26.wt, and A549 cell lines (*n* = 3). (**e**) Evaluation of anticancer efficacy on Hep G2 cells treated with DTX, PTX, PTXm, (Ac)FRRF, (Ac)FRRF-DTX NPs and de-Boc-DTX (*n* = 5 − 6). (**f**) Cellular uptake of RITC-PTX and RITC-(Ac)FRRF-DTX NPs in Hep G2 cells (Scale bar = 50 μm). (**g**) Data quantifying the fluorescence intensity over time of (Ac)FRRF-DTX NPs and PTX (*n* = 3). (**h**) Fluorescent microscopy imaging observing cell death in Hep G2 cells treated with PTX, PTXm and (Ac)FRRF-DTX NPs (Scale bar = 50 μm). (**i**) Quantification of fluorescence intensity from nucleic acid staining using ImageJ software (*n* = 5). (**j**) microtubule binding of PTX, PTXm and (Ac)FRRF-DTX NPs (Scale bar = 50 μm). (**k**) Quantification of fluorescence intensity from Microtubule Cytoskeleton Dye using ImageJ software (*n* = 5). (**l**) Confocal images of (Ac)FRRF-DTX NPs (Scale bar = 10 μm). *p < 0.05, **p < 0.01 and ***p < 0.001

We hypothesized that (Ac)FRRF-DTX NPs, incorporating the FRR peptide, would undergo selective nanoconversion to PTXm due to their elevated specificity for cathepsin B. To validate this strategy, HPLC and LC-MS analyses were conducted to confirm whether (Ac)FRRF-DTX underwent cathepsin B-mediated nanoconversion, leading to the formation of PTXm [37–39]. In the absence of cathepsin B, (Ac)FRRF-DTX remained unchanged over time (Fig. 4a). When (Ac)FRRF-DTX NPs were treated with cathepsin B at a concentration of 50 µg/mL in MES buffer, no significant changes were observed in the media composition during the first 3 h. However, after this interval, a noticeable reduction in (Ac)FRRF-DTX levels was observed, coinciding with an increase in PTXm. At 48 h, complete nanoconversion of (Ac)FRRF-DTX to PTXm was observed (Fig. 4b). LC-MS analysis of (Ac)FRRF-DTX with cathepsin B after 72 h corresponded to the predicted molecular weight of PTXm (Fig. 4c). This demonstrates that the peptide segment RR of (Ac)FRRF-DTX was gradually cleaved by cathepsin B, which was overexpressed in cancer cells, leading to its nanoconversion to PTXm.

We examined the expression levels of cathepsin B in selected cancer cell lines. Most cancer cell lines showed higher levels of cathepsin B compared to the normal cell line HDFa, with Hep G2 showing the highest expression: Hep G2 (5961.33 ± 71.18 a.u.), CT26.wt (5453.73 ± 156.87 a.u.), and A549 (3118.30 ± 145.20 a.u.) vs. HDFa (1917.03 ± 25.41 a.u.) (Fig. 4d) [40–42]. Based on these results, subsequent experiments were conducted using Hep G2 cancer cells. When comparing cytotoxicity in Hep G2 cells at 500 µM relative to the untreated group, (Ac)FRRF-DTX showed a cell viability of 21.2 ± 3.1% (IC_50_ = 229.4 µM), indicating significantly higher cytotoxicity than the other groups: DTX at 57.9 ± 8.9% (IC_50_ = 316.4 µM), PTX at 55.5 ± 4.9% (IC_50_ = 322.5 µM), PTXm at 40.9 ± 10.9% (IC_50_ = 213.5 µM), (Ac)FRRF at 84.1 ± 6.6% (IC_50_ = 3083 µM), and de-Boc-DTX at 75.3 ± 5.4% (IC_50_ = 1777 µM) (Fig. 4e). Since DTX and PTX are well-known for their high cytotoxicity against cancer cells, we anticipate that these results may have been influenced by solubility differences, as the experiments were conducted without DMSO; de-Boc-DTX also shows low water solubility and cytotoxic effects similar to those of DTX. Therefore, only the water-soluble (Ac)FRRF-DTX NPs were able to fully exhibit their cytotoxicity against Hep G2 cancer cells, in contrast to the insoluble drugs DTX and PTX. To clarify this expectation, when the solubility of DTX and PTX was enhanced using 3% DMSO, pronounced cytotoxicity was observed. Evaluating the cytotoxicity of DTX and PTX at 500 µM in a 3% DMSO solution in Hep G2 cells relative to the untreated group, DTX showed a cell viability of 24.9 ± 4.6% (vs. 57.9 ± 8.9% without DMSO), and PTX showed a cell viability of 27.4 ± 4.5% (vs. 55.5 ± 4.9% without DMSO) (Fig. S3). These results suggest that cellular toxicity is inherently limited by the solubility differences of poorly water-soluble compounds like DTX and PTX. However, (Ac)FRRF-DTX NPs not only exhibit high water solubility but also become the active form, PTXm, in response to the tumor environment through cathepsin B-mediated nanoconversion, allowing for more significant cytotoxicity in Hep G2 cancer cells at high concentrations compared to DTX, PTX, and PTXm.

Further, fluorescence microscopy was used to assess the cellular uptake of (Ac)FRRF-DTX NPs, after incubation of Hep G2 cells with the RITC-labeled PTX (RITC-PTX) and (Ac)FRRF-DTX (RITC-(Ac)FRRF-DTX). RITC reacts with the hydroxyl groups of PTX or (Ac)FRRF-DTX to form thiocarbamate bonds, resulting in stable covalent attachment. Initially, observations were limited to blue fluorescence (DAPI) in RITC-PTX. However, the intensity of the red fluorescent region was observed at same period in RITC-(Ac)FRRF-DTX. Over time, both showed incrementally increasing red fluorescence, although this was more pronounced in RITC-(Ac)FRRF-DTX. After 4 h, the red fluorescence completely overlapped with the blue area, indicating that the (Ac)FRRF-DTX NPs had entered the cells (Fig. 4f). Quantitative analysis of fluorescence intensity over time revealed that RITC-(Ac)FRRF-DTX reached saturation at 4 h, whereas RITC-PTX did not reach saturation even after 8 h. Furthermore, at 8 h, the fluorescence intensity for RITC-(Ac)FRRF-DTX (439.85 ± 314.14 a.u.) was higher than that of RITC-PTX (390.04 ± 10.29 a.u.) (Fig. 4g). This indicated that (Ac)FRRF-DTX NPs had a higher cell uptake compared to PTX. As anticipated from the cytotoxicity results, when Hep G2 cells were treated at a concentration of 100 µM, (Ac)FRRF-DTX NPs exhibited toxicity levels similar to those of PTX and PTXm, as assessed by nucleic acid staining and quantified using ImageJ (U.S. National Institutes of Health) (Fig. 4h and i). These results were consistent with the previous cytotoxicity findings and further confirmed the outcomes of the cytotoxicity assay. Additionally, to verify whether it was involved in the microtubule formation process, as was well known for taxol-based active compounds, a microtubule-binding kit (BioTracker™ 488 Green Microtubule Cytoskeleton Dye, Sigma-Aldrich, MO, USA) was used to treat cells with PTX, PTXm, and (Ac)FRRF-DTX, followed by imaging experiments using FITC fluorescence microscopy (ECLIPSE Ti2 Series, Nikon, Japan) and quantification through ImageJ (U.S. National Institutes of Health) (Fig. 4j and k) [43, 44]. In contrast to the well-organized microtubule structure observed in the control group, microtubule structures appeared indistinct in the groups treated with PTX, PTXm, and (Ac)FRRF-DTX NPs. This result means that PTXm, similar to the taxol-based chemotherapeutic PTX, acts as a microtubule depolymerization inhibitor and that PTXm was generated within (Ac)FRRF-DTX NPs through cathepsin B-mediated nanoconversion.

To further investigate the intracellular uptake of (Ac)FRRF-DTX in Hep G2 cancer cells, confocal imaging was conducted using RITC-(Ac)FRRF-DTX, which was labeled with RITC fluorescent dye. After treatment, the localization pattern was found to be more similar to that of Phalloidin than to DAPI, suggesting that (Ac)FRRF-DTX NPs were distributed within the cytoplasm, where microtubules are present, rather than in the nucleus. These findings suggest that (Ac)FRRF-DTX is internalized by the cells and binds to the abundant microtubules in the cytoplasm, thereby inhibiting cancer cell division in a manner similar to that of taxol (Fig. 4l). Therefore, based on these results and the in silico study, which showed that PTXm formed through nanoconversion of (Ac)FRRF-DTX NPs has structural, electrical, and docking properties similar to PTX and exhibited substantially similar biological activity, the cytotoxicity of the (Ac)FRRF-DTX NPs may be attributed to a mechanism similar to that of PTX.

### Targeting and biodistribution of (Ac)FRRF-DTX NPs

**Fig. 5:**
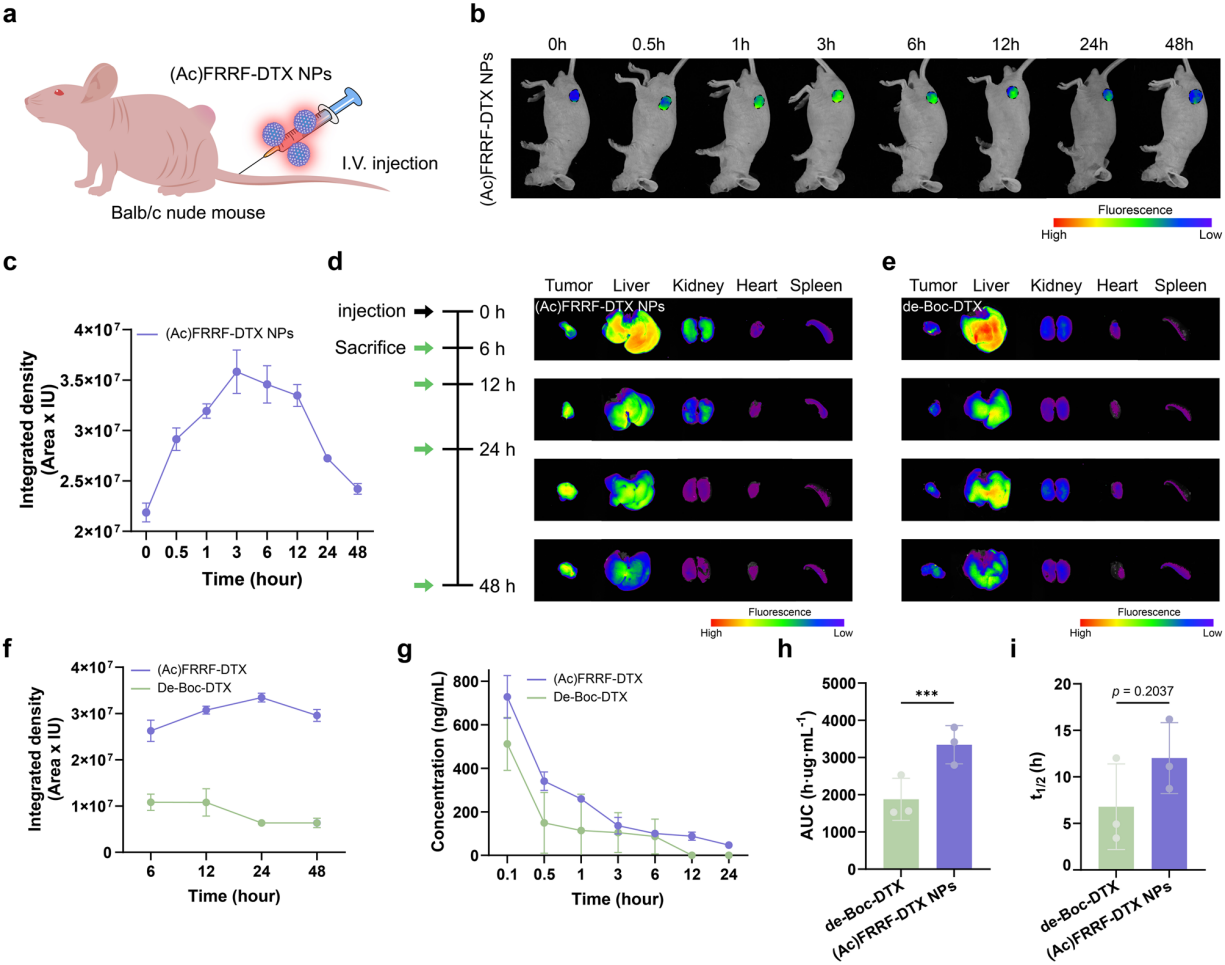
In vivo biodistribution and retention level in the bloodstream of (Ac)FRRF-DTX NPs. (**a**) Scheme describing the in vivo distribution study of (Ac)FRRF-DTX NPs using BALB/c nude mice. (**b**) In vivo distribution over time of RITC-(Ac)FRRF-DTX NPs administered via tail vein injection in BALB/c nude mice. (**c**) Quantified graphs depicting fluorescent intensity in tumor regions (*n* = 3). Images showing the measurement of fluorescence intensity from hourly extractions of organs and tumors in mice treated with RITC-(Ac)FRRF-DTX NPs (**d**) and treated with RITC-de-Boc-DTX (**e**). (**f**) Quantified graphs depicting fluorescent intensity in tumor tissues over time (*n* = 3). (**g**) The concentration in the bloodstream of Sprague-Dawley rats treated with RITC-(Ac)FRRF-DTX NPs and RITC-de-Boc-DTX administered via tail vein injection (*n* = 3). The AUC (h) and half-life (i) (*n* = 3). *p < 0.05, **p < 0.01 and ***p < 0.001

To explore in vivo ability of (Ac)FRRF-DTX NPs to accumulate in tumors, BALB/c nude mice bearing Hep G2 tumors were used as an antitumor xenograft model. To confirm the accumulation of (Ac)FRRF-DTX NPs at the tumor sites where Hep G2 cancer cells were implanted, RITC-labeled (Ac)FRRF-DTX NPs were used in biodistribution studies (Fig. 5a). RITC-(Ac)FRRF-DTX NPs were administered intravenously via the tail vein of mice at a concentration of 20 mg/kg. Imaging was performed at designated time points (0, 0.5, 1, 3, 6, 12, 24 and 48 h) to evaluate the accumulation in tumor tissues. Also, the fluorescence intensity in nude mice was captured using a precision fluorescence analyzer (FOBI, CELLGENTEK, Republic of Korea). Following administration, an increase in fluorescence was observed over time at the tumor tissue sites, steadily rising until 3 h. The fluorescence intensity at the tumor sites remained high at 12 h and gradually decreased over the subsequent 48 h (Fig. 5b). Quantitative analysis revealed that the fluorescence intensity at 3 h, which was the highest observed, increased approximately 1.64-fold compared to that at 0 h. The intensity was maintained to some extent up to 12 h, after which it decreased, consistent with the biodistribution images (Fig. 5c). To compare the biodistribution of (Ac)FRRF-DTX NPs and de-Boc-DTX, RITC-(Ac)FRRF-DTX NPs and RITC-de-Boc-DTX were administered via a single intravenous injection through the tail vein of mice. Tumor tissues and major organs, including the liver, kidney, heart, and spleen, were excised at designated time points (0, 6, 12, 24, and 48 h) and analyzed for fluorescence intensity to determine the accumulation and biodistribution of the two compounds (Fig. 5d and e). In the group treated with RITC-(Ac)FRRF-DTX NPs, fluorescence intensity was consistently strong specifically within the tumor tissue rather than in other organs, whereas in the group treated with RITC-de-Boc-DTX exhibited weak fluorescence intensity from the outset and progressively diminished, reaching only about one-fifth of the intensity observed in the RITC-(Ac)FRRF-DTX NPs group after 48 h (Fig. 5f). This suggests that selective accumulation in the tumor was achieved with (Ac)FRRF-DTX NPs. Additionally, the retention level in the bloodstream of (Ac)FRRF-DTX NPs and de-Boc-DTX were examined by administering RITC-(Ac)FRRF-DTX NPs and RITC-de-Boc-DTX via tail vein injection in Sprague-Dawley rats. The group treated with RITC-(Ac)FRRF-DTX NPs showed a higher retention level in the bloodstream over time, with a 1.78 times higher AUC and a 1.77 times longer half-life compared to the group treated with RITC-de-Boc-DTX (Fig. 5g − i). We expect that modifying de-Boc-DTX (6) to (Ac)FRRF-DTX NPs, which form stable self-assembled nanoparticles, significantly prolonged their circulation time in the bloodstream.

### Antitumor efficacy and systemic toxicity analysis

**Fig. 6:**
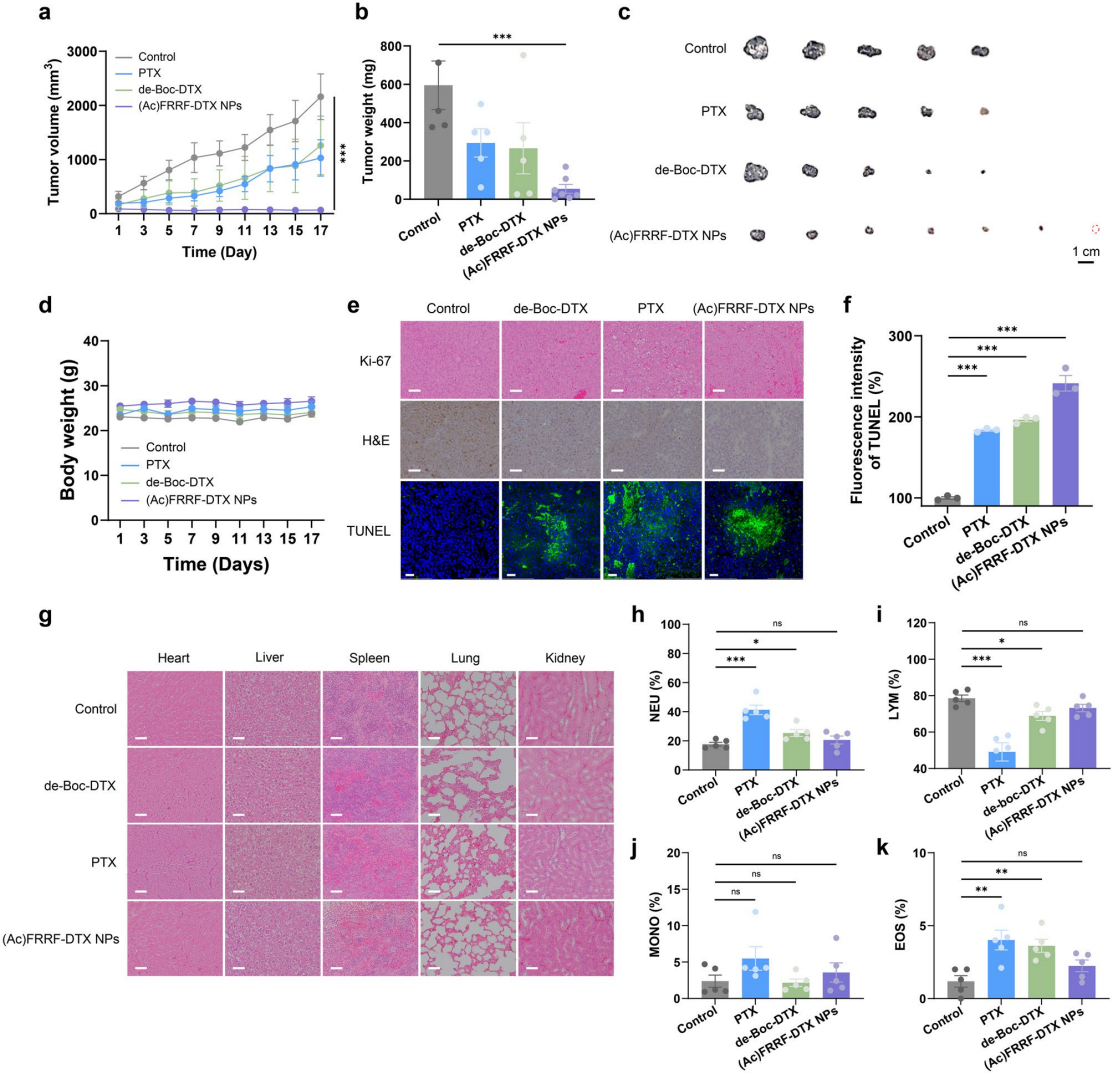
Anticancer efficacy evaluation. (**a**) Tumor growth curves for mice treated with different agents (PTX, de-Boc-DTX: 5 mg/kg, (Ac)FRRF-DTX NPs: 10 mg/kg) (*n* = 5–7). (**b**) Average tumor weights for each group after 17 days of treatment (*n* = 5–7). (**c**) Images depicting tumor sizes extracted from the backs of mice after 17 days of treatment (*n* = 5–7). (**d**) Mouse weights measured during the 17 days anticancer treatment (*n* = 5–7). (**e**) Images of tumor slices collected at the experimental endpoint stained with H&E, Ki-67, and TUNEL assay (Scale bar = 50 μm). (**f**) quantified graph of TUNEL staining (*n* = 3). (**g**) Images of H&E staining in the major organs (heart, liver, spleen, lung, kidney) after the in vivo antitumor experiment (Scale bar = 50 μm). White blood cells count between PTX, de-Boc-DTX, (Ac)FRRF-DTX NPs, and the normal control groups Neutrophils (NEU) (**h**), Lymphocytes (LYM) (**i**), Monocytes (MONO) (**j**), and Eosinophils (EOS) (**k**) (*n* = 5). Each group with “ns” indicates no significant difference. *p < 0.05, **p < 0.01 and ***p < 0.001

To validate the in vivo antitumor efficacy of nanoconverted (Ac)FRRF-DTX NPs, Hep G2 cell xenografts (1.0 × 10^7^ cells) were implanted into the backs of 6-week-old male BALB/c nude mice (Orient Bio, Seongnam, Republic of Korea). The in vivo antitumor evaluation commenced once the tumors reached a size of 50–100 mm^3^. Treatments were administered over a 17 days period, with injections conducted at 3 day intervals. The substances administered were de-Boc-DTX (5 mg/kg), PTX (5 mg/kg), (Ac)FRRF-DTX NPs (10 mg/kg; based on 5 mg/kg of de-Boc-DTX), and saline as control. PTX, a widely used FDA-approved drug, was selected as the control in this study to represent DTX, PTX, and PTXm, which exhibit similar cytotoxicity. After 17 days, tumor sizes in the PTX and de-Boc-DTX groups were measured at 1029.79 ± 752.19 mm^3^ and 1262.19 ± 1209.27 mm^3^, respectively, representing approximately half the size of the control group, whose tumor size was 2161.12 ± 946.71 mm^3^. In contrast, the group treated with (Ac)FRRF-DTX NPs exhibited no significant increase in tumor size, measuring just 66.41 ± 69.54 mm^3^ after the same period, which is approximately one-thirtieth the size of the control group (Fig. 6a). While PTX and de-Boc-DTX demonstrated mild tumor suppression—similar, though not identical, to their in vitro results—(Ac)FRRF-DTX NPs exhibited exceptional cytotoxicity in animal models. Additionally, after 17 days of the in vivo efficacy evaluation, tumors were excised and weighed. The average tumor weights were as follows: Control group, 595.28 ± 282.98 mg; PTX group, 293.97 ± 164.22 mg; de-Boc-DTX group, 265.94 ± 298.69 mg; and (Ac)FRRF-DTX NPs group, 54.22 ± 62.01 mg (Fig. 6b). Consistent with the size measurements, the group treated with (Ac)FRRF-DTX NPs exhibited a significant reduction in tumor weight, approximately one-tenth that of the control group. The difference in tumor size was also visually apparent (Fig. 6c). These highlights the potent tumor-suppressive effect of (Ac)FRRF-DTX NPs, as observed and quantitatively confirmed in vivo. Additionally, the groups treated with PTX, de-Boc-DTX, and (Ac)FRRF-DTX NPs exhibited no significant changes in body weight, similar to the control group. These compounds effectively target tumors without inducing short-term toxicity (Fig. 6d).

To histologically confirm the high levels of cell apoptosis in the excised tumor tissues, H&E and Ki-67 staining were performed. In H&E staining, the tissues treated with (Ac)FRRF-DTX NPs exhibited the highest number of abnormal cellular morphologies. Consistently, in Ki-67 staining, a marker used to assess the proliferation index of metastatic tumor cells, the (Ac)FRRF-DTX NPs group exhibited a markedly reduced presence of brown-stained traces indicative of cancer cell proliferation, in contrast to the extensive brown staining observed in the control group. Finally, TUNEL fluorescent staining revealed that the group treated with (Ac)FRRF-DTX NPs exhibited the strongest TUNEL fluorescence signals, consistent with previous results, confirming that nanoconversion effectively facilitates DNA fragmentation during apoptosis (Fig. 6e). Additionally, the quantitative fluorescence intensities of TUNEL staining, analyzed using ImageJ (U.S. National Institutes of Health), were measured as 183.71 ± 2.32% for PTX, 196.50 ± 4.25% for de-Boc-DTX, and 241.42 ± 16.53% for (Ac)FRRF-DTX NPs. The experimental groups exhibited intensities exceeding twice those of the control group, with (Ac)FRRF-DTX NPs demonstrating the highest intensity (Fig. 6f). Finally, to evaluate safety, histological assessments of toxicity were conducted on the major organs (heart, liver, spleen, lungs, and kidney) of mice from each group. Similar to the control groups, no significant toxicity was observed in the groups treated with de-Boc-DTX, PTX, or (Ac)FRRF-DTX NPs (Fig. 6g). To further evaluate overall toxicity, blood chemistry analyses of red blood cells (RBCs) and white blood cells (WBCs) were performed. The normal mice received a single intravenous administration via the tail vein of saline as control, de-Boc-DTX (1.5 mg/kg), PTX (1.5 mg/kg), or (Ac)FRRF-DTX NPs (3 mg/kg). Seven days post-administration, blood samples were collected via cardiac puncture and analyzed using a hematology analyzer (Sysmex XN series, Sysmex, Japan). The HGB, HCT, MCV, MCH, and MCHC showed no significant changes across all groups (Fig. S4). As previously reported, the group treated with PTX exhibited alterations in NEU, LYM and EOS concentrations [45, 46]. In contrast, the group treated with (Ac)FRRF-DTX NPs exhibited no significant changes under the same conditions, as assessed by blood chemistry (Fig. 6h − 6k). The formation of (Ac)FRRF-DTX NPs effectively reduced the known in vivo toxicity associated with taxol-based compounds as assessed by blood chemistry analysis. Moreover, blood toxicity markers for liver and kidney function, including AST, ALP, LDH, LAC, TP, Alb, A/G ratio, BUN, and Crea, showed no significant changes. Overall, (Ac)FRRF-DTX NPs exhibited low systemic toxicity in vivo, further supporting their favorable safety profile (Fig. S5).

## Conclusion

This study successfully demonstrated the development of a novel peptide-drug conjugate nanoparticle, (Ac)FRRF-DTX (**5**), which undergoes tumor-selective nanoconversion mediated by cathepsin B, resulting in the formation of the paclitaxel mimic PTXm (**3**). The amphiphilic design of (Ac)FRRF-DTX (**5**) enables spontaneous self-assembly into nanoparticles, as confirmed by in silico molecular dynamics modeling, exhibiting enhanced solubility and stability. Additionally, the ability of PTXm (**3**), resulted by nanoconversion, was simulated the structural and funcational similarity of PTX (**2**).

Biological evaluations revealed that (Ac)FRRF-DTX nanoparticles ((Ac)FRRF-DTX NPs) exhibit selective cytotoxicity toward Hep G2 cancer cells while maintaining minimal toxicity in normal cells. This selectivity is facilitated by the cathepsin B-mediated nanoconversion of (Ac)FRRF-DTX (**5**). Moreover, (Ac)FRRF-DTX NPs inhibit microtubule formation, similar to conventional DTX (**1**) and PTX (**2**), but exhibit enhanced selectivity and potency. In vivo studies confirmed the exceptional antitumor efficacy of (Ac)FRRF-DTX NPs, exhibiting significant tumor suppression and minimal systemic toxicity through nanoconversion. Also, biodistribution studies revealed prolonged retention in tumor tissues and the bloodstream, confirming selective accumulation and reduced off-target effects associated with this nanoconversion approach.

Overall, this study establishes (Ac)FRRF-DTX nanoparticles ((Ac)FRRF-DTX NPs) as a promising therapeutic platform, offering improved solubility, tumor specificity, and potent antitumor activity through enzymatic nanoconversion. Our innovative nanoconversion approach provides a foundation for future development of peptide-drug conjugates and nanoparticle-based therapeutics for targeted cancer therapy. Notably, nanoconversion enhances the activity and specificity of derivatives with limited chemical structures that were previously challenging to deliver. Further applications of this novel nanoconversion strategy for other drugs are currently under investigation in our laboratory.

## Electronic supplementary material

Below is the link to the electronic supplementary material.

Supplementary Material 1

## Data Availability

Data will be made available on request.

## Abbreviations

(Ac)FRRF: Acetylated Phenylalanine-Arginine-Arginine-Phenylalanine
(Ac)FRRF-DTX: Acetylated Phenylalanine-Arginine-Arginine-Phenylalanine Docetaxel
A/G: Albumin/Globulin Ratio
Alb: Albumin
ALP: Alkaline Phosphatase
AST: Aspartate Aminotransferase
BUN: Blood Urea Nitrogen
Crea: Creatinine
DAPI: 4’,6-Diamidino-2-Phenylindole
DFT: Density Functional Theory
DOX: Doxorubicin
DTX: Docetaxel
EDCI: 1-Ethyl-3-(3-dimethylaminopropyl) carbodiimide
EOS: Eosinophils
FITC: Fluorescein Isothiocyanate
H&E: Hematoxylin and Eosin
HCT: Hematocrit
HGB: Hemoglobin
HOMO: Highest Occupied Molecular Orbital
Ki-67: A marker of cell proliferation
LAC: Lactate
LDH: Lactate Dehydrogenase
LUMO: Lowest Unoccupied Molecular Orbital
LYM: Lymphocytes
MCH: Mean Corpuscular Hemoglobin
MCHC: Mean Corpuscular Hemoglobin Concentration
MCV: Mean Corpuscular Volume
MES: 2-(N-morpholino)ethanesulfonic acid
MONO: Monocytes
NEU: Neutrophils
NHS: N-Hydroxysuccinimide
NPs: Nanoparticles
PDC: Peptide Drug Conjugate
PFA: Paraformaldehyde
PLT: Platelets
PTX: Paclitaxel
PTXm: Paclitaxel mimic
RBC: Red Blood Cells
RITC: Rhodamine B Isothiocyanate
TMS: Trimethylsaline
TP: Total Protein
TUNEL: Terminal deoxynucleotidyl transferase dUTP nick end labeling
WBC: White Blood Cells
