# Supplementary material for: Tumor-specific biochemical nanoconversion of self-assembled peptide-conjugated paclitaxel-docetaxel-based nanoparticles
Source: Nano Converg. 2025 Apr 26;12:20. doi: 10.1186/s40580-025-00487-0 (PMC12033163; doi:10.1186/s40580-025-00487-0)

Supplementary information

Tumor-specific biochemical nanoconversion of self-assembled peptide-conjugated paclitaxel-docetaxel-based nanoparticles

Hansol Lim[a, 1], Jae-Hyeon Lee[a, 1], So-Hyeon Park[a, 1], Jun-Hyuck Lee[a], Hyesu Jang[b], Seong-Bin Yang[a], Minho Seo[a], Seokwoo Lee[b*], Jooho Park[a,c*]

[a]Department of Applied Life Science, BK21 Program, Konkuk University, Chungju 27478, Republic of Korea

[b]College of Pharmacy, Chungnam National University, Daejeon 34134, Republic of Korea

[c]Department of Biomedical Chemistry, College of Biomedical and Health Science, Konkuk University, Chungju 27478, Republic of Korea

[1]These authors contributed equally to this work

[*]Corresponding author: Jooho Park (pkjhdn@kku.ac.kr), Seokwoo Lee (woolee@cnu.ac.kr)

1. Supplementary materials

All chemicals were of reagent-grade and were used as purchased. All reactions were performed under an inert atmosphere of dry nitrogen using distilled dry solvents. The reactions were monitored with TLC analysis using silica gel 60 F-254 thin layer plates. Compounds on the TLC plates were visualized under UV light and by spraying with either potassium permanganate or anisaldehyde solutions. Flash column chromatography was conducted on silica gel 60 (230–400 mesh). ^{1}H and ^{13}C NMR spectra were recorded on a Bruker Avance Neo 400 (400 MHz), Bruker Avance III 600 (600 MHz) or Bruker Avance III 600 MHz and Bruker Avance III HD 800 MHz spectrometer equipped with a 5 mm triple resonance inverse (TCI) Cryoprobe at 298 K if not noted otherwise. Chemical shifts are reported in ppm (δ) units relative to the undeuterated solvent as a reference peak (D_2O–d_2: 4.80 ppm/^{1}H NMR, CD_3OD–d_4: 3.30 ppm/^{1}H NMR, 49.00 ppm/^{13}C NMR). The following abbreviations are used to represent NMR peak multiplicities: s (singlet), d (doublet), t (triplet), m (multiplet), dd (doublet of doublets), dt (doublet of triplets), dq (doublet of quartets), td (triplet of doublets), and br (broad signal). High-resolution mass spectra (HRMS) were recorded using electrospray ionization (ESI) mass spectrometry on Sciex 1290 infinity II/TripleTOF 5600 plus.

Antibiotic antimycotic solution (100X), Bio Tracker^{TM} 488 Green Microtubule Cytoskeleton Dye, Cathepsin B from bovine spleen, Dimethyl sulfoxide (DMSO), Dulbecco's modified Eagle's medium (DMEM), 1-ethyl-3-(3-dimethylaminopropyl) carbodiimide (EDCI), dichloromethane (DCM), ether, phosphate-buffered saline (PBS), rhodamine B isothiocyanate (RITC), 2-(4-amidi-nophenyl)-6-indolecarbamidine dihydrochloride (DAPI), Tetramethylsilane (TMS), Dimethyl sulfoxide-d_6 (DMSO-d_6) were purchased from Sigma-Aldrich (St. Louis, MO, USA). Paraformaldehyde solution 4% in PBS (PFA), Pierce^{TM} BCA Protein Assay Kit were obtained from Thermo Fisher Scientific (Waltham, MA, USA). EZ-Cytox kit was purchased from DoGenBio (Seoul, Republic of Korea). Fetal bovine serum (FBS), 0.5% Trypsin-EDTA (10X) were obtained from Gibico (Waltham, MA, USA). Neutral buffered formalin (10%) was purchased from HuBenTech (Damyang, Republic of Korea).

2. Supplementary method

2-1. Synthesis of (Ac)FRRF-DTX (5) and PTXm (3)

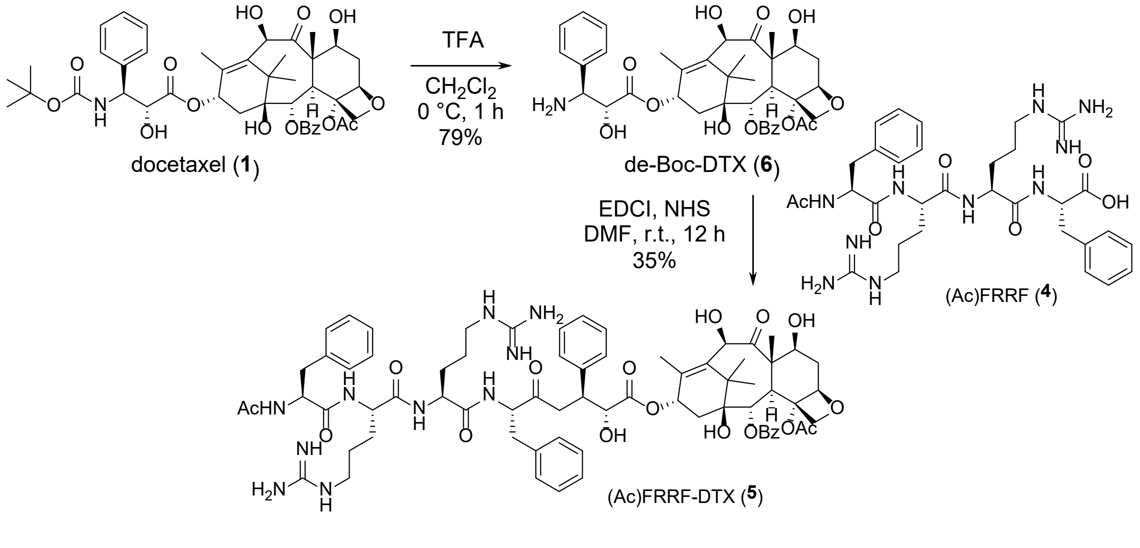

Scheme S1. Synthesis of (Ac)FRRF-DTX (**5**)

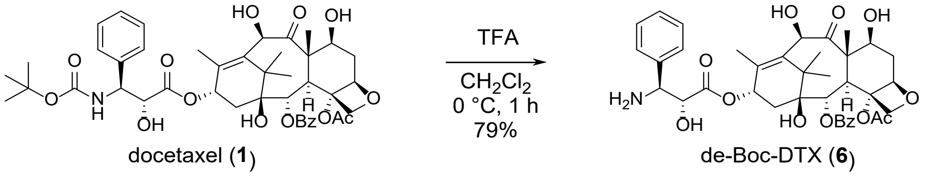

(2a*R*,4*S*,4a*S*,6*R*,9*S*,11*S*,12*S*,12a*R*,12b*S*)-12b-acetoxy-9-(((2*R*,3*S*)-3-amino-2-hydroxy-3-phenylpropanoyl)oxy)-4,6,11-trihydroxy-4a,8,13,13-tetramethyl-5-oxo-2a,3,4,4a,5,6,9,10,11,12,12a,12b-dodecahydro-1H-7,11-methanocyclodeca[3,4]benzo[1,2-b]oxet-12-yl benzoate (6): Docetaxel (200 mg, 0.24 mmol, 1.0 equiv) was dissolved in anhydrous CH_2Cl_2 (3 mL). The solution was cooled to 0 ºC in an ice bath and to this was added dropwise trifluoroacetic acid (TFA, 1mL). The reaction bottle was stirred for 1 h at the same temperature and the solvent was evaporated under vacuum. The obtained crude was dissolved in EtOAc (10 mL) and the organic phase washed saturated $NaHCO_3$ solution (10 mL) and H_2O (10 mL). The organic layer was dried over Na_2SO_4 and concentrated *in vacuo* to give a white solid. The crude product was recrystallized from CH_2Cl_2, diethyl ether to obtain compound **6** (134 mg, 79%) as a white solid.

TLC R_f = 0.4 (CH_2Cl_2:MeOH = 5:1, *v*/*v*)

^{1}H NMR (800 MHz, CD_3OD) δ 8.06 – 8.03 (m, 2H), 7.71 (d, *J* = 6.2 Hz, 1H), 7.61 (t, *J* = 7.3 Hz, 2H), 7.49 (d, *J* = 7.4 Hz, 4H), 7.37 (d, *J* = 6.9 Hz, 1H), 6.08 (t, *J* = 8.9 Hz, 1H), 5.59 (d, *J* = 7.2 Hz, 1H), 5.24 (s, 1H), 4.97 (dd, *J* = 9.6, 2.2 Hz, 1H), 4.42 (s, 1H), 4.19 (d, *J* = 4.8 Hz, 1H), 4.18 – 4.15 (m, 2H), 3.80 (d, *J* = 7.3 Hz, 1H), 2.44 (t, *J* = 5.7 Hz, 1H), 2.22 (s, 3H), 1.99 – 1.93 (m, 3H), 1.88 (d, *J* = 1.5 Hz, 3H), 1.84 (d, *J* = 3.0 Hz, 1H), 1.69 (s, 3H), 1.15 (s, 3H), 1.12 (s, 3H)

^{13}C NMR (200 MHz, CD_3OD) δ 211.0, 173.4, 171.7, 167.6, 138.9, 138.1, 134.7, 131.4, 131.1, 130.5, 130.3, 129.7, 128.9, 85.9, 82.3, 79.1, 77.5, 76.3, 75.7, 75.6, 72.7, 72.3, 59.5, 58.8, 47.9, 44.4, 37.4, 36.7, 26.9, 23.2, 21.5, 14.4, 10.4

HRMS (ESI): m/z calculated for $C_{38}H_{46}NO_{12}$ $[M+H]^+$ 708.3015, found 708.3015

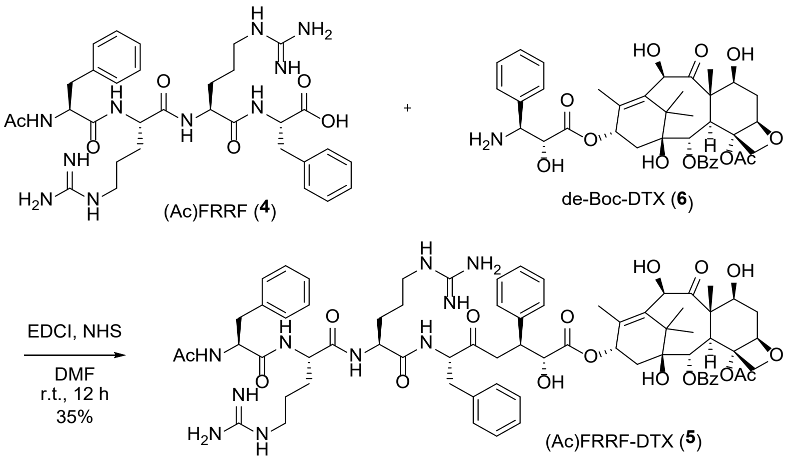

acetyl-*L*-phenylalanyl-*L*-arginyl-*L*-arginyl-*L*-phenylalanine ((Ac)FRRF, 4):

^{1}H NMR (600 MHz, D_2O) δ 7.35 – 7.32 (m, 4H), 7.30 – 7.23 (m, 6H), 4.69 – 4.66 (m, 1H), 4.52 (t, *J* = 7.5 Hz, 1H), 4.20 (t, *J* = 7.1 Hz, 2H), 3.24 (dd, *J* = 14.0, 5.0 Hz, 1H), 3.12 (dt, *J* = 30.4, 6.9 Hz, 4H), 3.04 – 3.00 (m, 3H), 1.96 (s, 3H), 1.74 – 1.38 (m, 8H)

^{13}C NMR (150 MHz, D_2O) δ 174.5, 174.0, 173.1, 172.9, 172.3, 162.9 (q, *J* = 35.5 Hz), 156.62, 156.59, 136.5, 136.1, 129.2, 129.1, 128.7, 128.6, 127.1, 127.0, 116.3 (q, *J* = 291.7 Hz), 55.2, 54.0, 53.1, 52.8, 40.5 (2C), 40.4 (2C), 36.9, 36.6, 28.21, 28.17, 24.3, 24.1, 21.5

(2a*R*,4*S*,4a*S*,6*R*,9*S*,11*S*,12*S*,12a*R*,12b*S*)-12b-acetoxy-9-(((4S,7S,10S,13S,16S,17R)-4,13-dibenzyl-7,10-bis(3-guanidinopropyl)-17-hydroxy-2,5,8,11,14-pentaoxo-16-phenyl-3,6,9,12,15-pentaazaoctadecan-18-oyl)oxy)-4,6,11-trihydroxy-4a,8,13,13-tetramethyl-5-oxo-2a,3,4,4a,5,6,9,10,11,12,12a,12b-dodecahydro-1*H*-7,11-methanocyclodeca[3,4]benzo[1,2-*b*]oxet-12-yl benzoate (5): To a solution of **3** (30 mg, 0.04 mmol, 1.5 equiv) and (Ac)FRRF **4** (18 mg, 0.03 mmol, 1.0 equiv) in DMF (2 mL), EDCI (14.9 mg, 0.08 mmol, 3.0 equiv) and *N*-hydrosuccinimide (NHS, 5.9 mg, 0.05 mmol, 2.0 equiv) were added at room temperature. The mixture was stirred at room temperature for 12 h. The solvent was concentrated under reduced pressure and the crude recrystallized from CH_2Cl_2/diethyl ether (1: 1, 20 mL) to obtain **5** (21 mg, 35%) as a white solid.

^{1}H NMR (800 MHz, CD_3OD) δ 8.13 – 8.11 (m, 2H), 7.67 – 7.64 (m, 1H), 7.57 (t, *J* = 7.8 Hz, 2H), 7.43 – 7.40 (m, 4H), 7.27 – 7.26 (m, 5H), 7.25 (dd, *J* = 5.4, 1.4 Hz, 5H), 7.18 (d, *J* = 1.5 Hz, 1H), 6.17 (td, *J* = 9.1, 1.7 Hz, 1H), 5.64 (d, *J* = 7.1 Hz, 1H), 5.42 (d, *J* = 5.1 Hz, 1H), 5.25 (s, 1H), 5.00 – 4.98 (m, 1H), 4.73 (dd, *J* = 9.9, 4.8 Hz, 1H), 4.63 (d, *J* = 5.2 Hz, 1H), 4.56 (dd, *J* = 9.5, 5.4 Hz, 2H), 4.40 – 4.37 (m, 2H), 4.31 – 4.28 (m, 3H), 4.21 – 4.17 (m, 3H), 3.87 (d, *J* = 7.1 Hz, 1H), 3.25 – 3.21 (m, 6H), 3.19 – 3.10 (m, 16H), 2.43 (td, *J* = 7.9, 3.2 Hz, 1H), 2.34 (s, 3H), 1.93 (s, 3H), 1.92 – 1.91 (m, 2H), 1.90 (s, 3H), 1.87 – 1.86 (m, 2H), 1.70 (s, 3H), 1.64 – 1.60 (m, 8H), 1.20 (s, 3H), 1.13 (s, 3H)

^{13}C NMR (200 MHz, CD_3OD) δ 211.1, 210.0, 177.5, 175.0, 174.5, 174.1, 173.9, 173.7, 173.6, 173.3, 172.8, 171.9, 167.7, 161.7, 158.7, 158.6, 158.5, 139.7, 139.2, 139.1, 138.4, 138.4, 138.3, 138.0, 134.6, 132.4, 131.5, 131.3, 131.1, 130.9, 130.5, 130.4, 130.2, 130.2, 130.0, 129.6, 129.5, 129.5, 129.1, 129.0, 128.5, 127.9, 127.9, 127.8, 127.4, 127.3, 85.9, 82.4, 79.3, 77.6, 76.5, 75.6, 75.1, 72.7, 72.7, 58.9, 56.9, 56.4, 56.3, 56.2, 54.6, 54.4, 54.2, 54.0, 47.9, 44.5, 43.4, 42.2, 42.0, 42.0, 42.0, 41.9, 41.9, 41.8, 41.7, 40.2, 38.8, 38.7, 38.6, 38.3, 37.5, 37.2, 36.9, 35.9, 30.1, 27.2, 26.3, 26.2, 25.8, 23.2, 22.5, 22.3, 21.7, 15.7, 14.5, 10.5

HRMS (ESI): m/z calculated for $C_{70}H_{90}N_{11}O_{17}$ $[M+2H]^{+2}$ 678.8292, found 678.8281

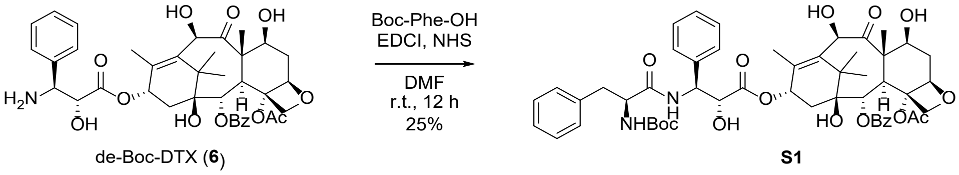

(2a*R*,4*S*,4a*S*,6*R*,9*S*,11*S*,12*S*,12a*R*,12b*S*)-12b-acetoxy-9-(((2*R*,3*S*)-3-((*S*)-2-((*tert*-butoxycarbonyl)amino)-3-phenylpropanamido)-2-hydroxy-3-phenylpropanoyl)oxy)-4,6,11-trihydroxy-4a,8,13,13-tetramethyl-5-oxo-2a,3,4,4a,5,6,9,10,11,12,12a,12b-dodecahydro-1*H*-7,11-methanocyclodeca[3,4]benzo[1,2-b]oxet-12-yl benzoate (S1): To a solution of **6** (57 mg, 0.08 mmol, 1.5 equiv) and Boc-l-phenylalanine (Boc-Phe-OH, 13 mg, 0.05 mmol, 1.0 equiv) in DMF (2 mL), EDCI (29 mg, 0.15 mmol, 3.0 equiv) and NHS (12 mg, 0.10 mmol, 2.0 equiv) were added at room temperature. The mixture was stirred at room temperature for 12 h. After the completion of the reaction, as monitored by HPLC, the reaction mixture was concentrated under reduced pressure. The crude mixture was purified by flash chromatography on silica gel (CH_2Cl_2:MeOH, 15:1, *v*/*v*) to obtain compound **S1** (19 mg, 25%) as a white solid.

TLC R_f = 0.4 (CH_2Cl_2:MeOH = 15:1, *v*/*v*)

^{1}H NMR (600 MHz, CD_3OD) δ 8.14 (d, *J* = 7.2 Hz, 2H), 7.65 (t, *J* = 7.3 Hz, 1H), 7.56 (t, *J* = 7.7 Hz, 2H), 7.44 – 7.38 (m, 4H), 7.30 (d, *J* = 7.2 Hz, 1H), 7.26 – 7.22 (m, 2H), 7.19 (dd, *J* = 11.6, 7.1 Hz, 3H), 6.20 (t, *J* = 8.9 Hz, 1H), 5.66 (d, *J* = 7.2 Hz, 1H), 5.47 (d, *J* = 4.1 Hz, 1H), 5.26 (s, 1H), 5.02 – 4.99 (m, 1H), 4.64 (d, *J* = 4.3 Hz, 1H), 4.38 (dd, *J* = 9.9, 4.7 Hz, 1H), 4.21 (d, *J* = 5.0 Hz, 2H), 3.89 (d, *J* = 7.2 Hz, 1H), 3.13 (dd, *J* = 14.0, 4.7 Hz, 1H), 2.79 (dd, *J* = 13.5, 10.5 Hz, 1H), 2.47 – 2.42 (m, 1H), 2.39 (s, 3H), 2.31 – 2.26 (m, 1H), 2.07 (dd, *J* = 15.3, 8.8 Hz, 1H), 1.88 (s, 3H), 1.87 – 1.77 (m, 2H), 1.70 (s, 3H), 1.35 (s, 9H), 1.20 (s, 3H), 1.13 (s, 3H)

^{13}C NMR (150 MHz, CD_3OD) δ 211.1, 174.3, 174.2, 171.9, 167.7, 157.7, 139.9, 139.3, 138.7, 137.9, 134.6, 131.3, 130.3, 129.7, 129.6, 129.4, 128.8, 128.4, 127.7, 86.0, 82.3, 80.8, 79.2, 77.6, 76.5,75.7, 74.7, 72.72, 72.67 (2C), 58.9, 57.4, 56.9, 47.9, 44.5, 38.9, 37.5, 37.0, 28.7, 27.2, 23.2, 21.7, 14.4, 10.5

HRMS (ESI): m/z calculated for $C_{52}H_{62}N_2O_{15}Na$ $[M+Na]^+$ 977.4042, found 977.4032

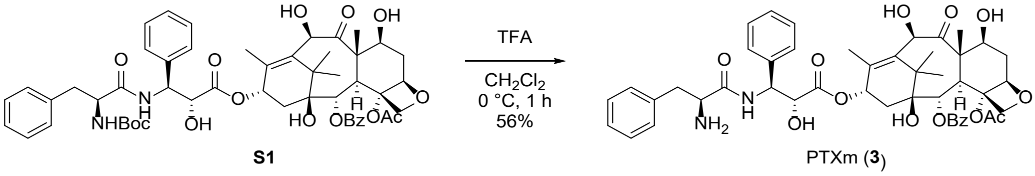

(2a*R*,4*S*,4a*S*,6*R*,9*S*,11*S*,12*S*,12a*R*,12b*S*)-12b-acetoxy-9-(((2*R*,3*S*)-3-((*S*)-2-amino-3-phenylpropanamido)-2-hydroxy-3-phenylpropanoyl)oxy)-4,6,11-trihydroxy-4a,8,13,13-tetramethyl-5-oxo-2a,3,4,4a,5,6,9,10,11,12,12a,12b-dodecahydro-1*H*-7,11-methanocyclodeca[3,4]benzo[1,2-b]oxet-12-yl benzoate (3): To a solution of **S1** (19 mg, 0.02 mmol, 1.0 equiv) in CH_2Cl_2 (0.3 mL), TFA (0.1 mL) was added at 0°C, and the mixture was stirred at the same temperature for 1 h. After the completion of the reaction, as monitored by HPLC, the reaction mixture was concentrated under reduced pressure. The crude mixture was purified by flash chromatography on silica gel (CH_2Cl_2:MeOH, 10:1, *v*/*v*) to obtain compound **3** (9.6 mg, 56%) as a white wax.

TLC R_f = 0.4 (CH_2Cl_2:MeOH = 10:1, *v*/*v*)

^{1}**H NMR** (600 MHz, CD_3OD) δ 8.12 (dd, *J* = 8.3, 1.1 Hz, 2H), 7.67 – 7.64 (m, 1H), 7.56 (t, *J* = 7.8 Hz, 2H), 7.38 (d, *J* = 4.7 Hz, 4H), 7.31 – 7.29 (m, 1H), 7.25 (d, *J* = 6.8 Hz, 2H), 7.21 – 7.19 (m, 3H), 6.18 (t, *J* = 9.7 Hz, 1H), 5.66 (d, *J* = 7.2 Hz, 1H), 5.46 (d, *J* = 4.2 Hz, 1H), 5.26 (s, 1H), 4.99 (dd, *J* = 9.6, 2.0 Hz, 1H), 4.61 (d, *J* = 4.2 Hz, 1H), 4.20 (d, *J* = 1.8 Hz, 2H), 3.88 (d, *J* = 7.2 Hz, 1H), 3.68 (dd, *J* = 8.3, 4.6 Hz, 1H), 3.09 (dd, *J* = 13.6, 4.6 Hz, 1H), 2.76 (dd, *J* = 13.6, 8.3 Hz, 1H), 2.46 – 2.41 (m, 1H), 2.36 (s, 3H), 2.29 (dd, *J* = 15.4, 9.5 Hz, 1H), 2.21 – 2.17 (m, 1H), 2.09 – 2.06 (m, 1H), 1.88 (d, *J* = 1.2 Hz, 3H), 1.85 – 1.81 (m, 1H), 1.70 (s, 3H), 1.19 (s, 3H), 1.13 (s, 3H)

^{13}**C NMR** (150 MHz, CD_3OD) δ 211.1, 176.5, 174.4, 172.0, 167.7, 140.0, 139.2, 138.8, 138.0, 134.6, 131.5, 131.2, 130.5, 129.7, 129.67, 129.64 (2C), 128.9, 128.4, 127.8, 85.9, 82.4, 79.2, 77.6, 76.5, 75.7, 74.8, 72.7, 58.9, 57.3, 56.7, 47.9, 44.6, 42.0, 37.5, 36.9, 27.7, 231, 21.7, 14.4, 10.5

HRMS (ESI): m/z calculated for $C_{47}H_{55}N_2O_{13}$ $[M+H]^+$ 855.3699, found 855.3690

2-2. Synthesis of RITC-labeled de-Boc-DTX, PTX and (Ac)FRRF-DTX

(Ac)FRRF-DTX (10 mg, 7.4 μmol, 1.0 equiv) was dissolved in 500 μL of DW, and separately, rhodamine B isothiocyanate (RITC, 1 mg, 1.9 μmol, 0.26 equiv) was dissolved in another 500 μL of DW. The two solutions were mixed and stirred at a low speed (600 rpm, RT) for 24 h. After the reaction, the solution was freeze-dried for 2 days with the addition of 10 mL of DW. Following freeze-drying, RITC-labeled RITC-(Ac)FRRF-DTX powder was gently precipitated by centrifugation (12000 rpm, 4 min, RT) in a solvent mixture of dichloromethane (DCM) and ether (1:1, v/v). Residual solvents were removed using a rotary evaporator, and the RITC-(Ac)FRRF-DTX (5.5 mg, 50%) was obtain as a purple powder after freeze-drying for 2 days. RITC-de-Boc-DTX was synthesized and purified using the same method as de-Boc-DTX (10 mg, 14 μmol, 1.0 equiv) and rhodamine B isothiocyanate (RITC, 1 mg, 1.9 μmol, 0.14 equiv). Following purification, RITC-de-Boc-DTX (4 mg, 36.36%) was obtained as a purple powder after freeze-drying for 2 days. RITC-PTX was synthesized using PTX (5 mg, 0.5 μmol, 1.0 equiv) and rhodamine B isothiocyanate (RITC, 10 mg, 19 μmol, 3.26 equiv), which were stirred together for overnight. Subsequent purification was performed continuously using distilled water (DW). After purification, RITC-PTX (0.5 mg, 3.33%) was obtained as a purple powder following freeze-drying for 2 days.

2-3. Microtubule docking

The molecular structures of PTX and PTXm were illustrated using Chemdraw Professional 20.1.1.125 (PerkinElmer Inc.). Molecular docking was conducted with the AMDock (Assisted Molecular Docking) software, targeting the microtubule protein (PDB: 5M50), and utilizing the CHARMm (Chemistry at Harvard Molecule Mechanics) force field. The binding site was identified based on the active site of the ligand present in the existing PDB. Among the 10 recorded poses, the most optimal one was chosen and visualized using the PyMOL program (Version 2.5.0; Schrödinger, LLC). To explore the interaction profile of the docked molecules, they were also displayed using discovery studio's 2D plot program.

2-4. Molecular energy calculations

The Density Functional Theory (DFT) calculations were performed using DMol^3 program in Discovery Studio 2022 software for HOMO and LUMO energy. The Perdew–Wang (PWC) local density approximation (LDA) exchange–correlation functional was applied, and a double numerical plus polarization (DNP) basis set was employed.

The Nonbond List Radius was set to the default value of 14 Ångströms, and for Electrostatics calculations, Kappa was set to 0.34, and Order was set to 4. The final entropy evaluation was conducted at a temperature of 298.15 K, following the default settings of Discovery Studio 2022 for the calculation method.

Table S1. The HOMO, LUMO, electrostatic, and free energy differences between PTX and PTXm.

Compound	HOMO (Ha)	LUMO (Ha)	Electrostatic energy (Kcal/mol)[a]	Free energy (Kcal/mol)[a]
PTX	-0.179039	-0.111908	-58.1299	1289.89398
PTXm	-0.186338	-0.111192	-72.71357	1606.68411

[a] 1 Ha = 627.509391 Kcal/mol.

Table S2. Cartesian coordinates of the structures

PTX (**2**)

Atom	X	Y	Z	Atom	X	Y	Z
C	126.6705	-17.5204	-0.663	O	129.3661	-19.3517	0.7458
C	127.231	-16.246	-0.1787	C	130.6108	-18.4863	0.971
C	126.6692	-14.9818	-0.6577	O	130.4669	-17.5091	1.7074
C	127.1953	-13.8211	-0.2661	C	132.0408	-18.6571	0.4318
C	128.3415	-13.8032	0.6434	H	127.4611	-21.2335	1.2826
C	128.8481	-14.9505	1.0862	H	130.6023	-21.4028	1.5529
C	128.2621	-16.2247	0.6527	H	125.817	-14.9895	-1.3373
O	125.8039	-17.4399	-1.5275	H	126.7707	-12.8833	-0.6242
O	127.1546	-18.7955	-0.1405	H	128.7779	-12.8547	0.9563
C	125.0166	-20.2091	-0.8583	H	129.6969	-14.9446	1.7699
C	126.7178	-20.1629	-0.6861	H	128.6796	-17.1628	1.0186
C	127.6967	-21.2702	0.2094	H	127.1069	-20.1996	-1.7037
C	129.2751	-20.6799	0.0001	H	129.6214	-21.2601	-2.1098
C	130.0018	-20.5964	-1.3332	H	130.1133	-19.5902	-1.7371
O	131.2008	-21.1195	-0.5718	H	130.2432	-23.1299	-0.9296
C	130.3386	-21.5868	0.5082	H	131.0088	-23.5863	0.6115
C	130.1855	-23.037	0.1549	H	128.7592	-23.5452	1.6852
C	128.8964	-23.6622	0.6102	H	124.6798	-24.0748	1.2775
C	127.717	-22.97	-0.1987	H	122.5826	-20.3866	-0.459
C	126.4702	-23.9495	0.0839	H	124.0984	-18.5473	0.4143
C	124.9541	-23.7642	0.2694	H	124.8262	-19.8462	1.3903
C	124.3153	-22.3948	-0.0236	H	122.8997	-22.8323	-2.4186
C	123.5132	-21.7361	0.9091	H	123.1028	-21.1827	-3.0546
C	122.9117	-20.3098	0.5773	H	122.2771	-21.4415	-1.4993
C	124.2388	-19.6249	0.4992	H	124.8831	-23.174	-2.854
C	124.4817	-21.6688	-1.3852	H	126.3264	-22.3246	-2.2521
O	126.7546	-25.1491	0.0807	H	125.2237	-21.5107	-3.3874
C	123.0784	-21.7911	-2.1505	H	122.5796	-21.5972	2.8301
C	125.2848	-22.2071	-2.5511	H	124.1631	-22.4095	2.8341
C	123.2268	-22.2878	2.2894	H	122.731	-23.2543	2.1992
C	128.1555	-23.2946	-1.6696	H	127.4295	-22.876	-2.3664
O	129.0172	-25.0433	0.2501	H	128.2064	-24.3751	-1.8041
O	124.6987	-19.3483	-1.9292	H	129.1359	-22.8584	-1.8616
O	121.7072	-19.8284	1.3177	H	129.1449	-25.1248	-0.7889
C	121.1837	-18.7209	0.4681	H	125.0267	-18.376	-1.7066
C	119.6593	-18.3054	0.3025	H	119.5084	-18.1261	-0.762
C	118.4505	-19.2761	0.7708	H	118.5025	-19.376	1.855
O	121.9013	-18.2788	-0.4383	H	120.6683	-20.7307	-0.3152
C	118.5082	-20.7578	0.1336	H	120.7852	-22.9835	-1.1406
C	119.7623	-21.3361	-0.3409	H	118.6967	-24.428	-1.2443
C	119.8294	-22.5821	-0.8039	H	116.5803	-23.5108	-0.4968
C	118.6418	-23.4082	-0.8634	H	116.4767	-21.156	0.4302
C	117.4866	-22.9072	-0.448	H	119.7436	-17.129	1.9608
C	117.4312	-21.5444	0.0751	H	113.276	-19.8473	0.8596
O	119.4935	-17.0414	0.9448	H	111.2585	-18.71	0.1772
C	115.8673	-18.949	0.7135	H	111.3884	-16.3432	-0.7749
C	114.6542	-18.1999	0.2934	H	113.5371	-15.2577	-1.0232
C	113.3387	-18.8428	0.441	H	115.6949	-16.4794	-0.3484
C	112.2243	-18.216	0.0709	H	117.2268	-17.6182	-0.102
C	112.2992	-16.8635	-0.4785	H	122.6016	-24.2856	1.1216
C	113.4841	-16.2675	-0.6163	H	121.9947	-25.956	1.0308
C	114.7276	-16.9655	-0.2214	H	121.2719	-24.6898	0.01
O	115.6721	-20.0089	1.3253	H	132.0856	-19.5387	-0.2077
N	117.1323	-18.5451	0.4242	H	132.7321	-18.7784	1.2658
O	124.5161	-24.7975	-0.6865	H	132.3197	-17.7756	-0.1456
C	123.1945	-25.354	-0.6236	H	132.0856	-19.5387	-0.2077
O	122.8882	-26.2062	-1.4638	H	132.7321	-18.7784	1.2658
C	122.1975	-25.0506	0.4587	H	132.3197	-17.7756	-0.1456

PTXm (**3**)

Atom	X	Y	Z	Atom	X	Y	Z
C	82.6674	-17.9639	0.014	C	98.3315	-22.8997	0.3284
O	82.5384	-18.5927	-1.0433	H	93.1814	-23.6993	1.3179
N	82.7569	-15.5862	-0.2102	H	95.9884	-25.0569	1.5503
C	81.8686	-16.6504	0.2162	C	82.4659	-14.2889	-0.033
C	80.5452	-16.488	-0.6475	C	83.4371	-13.2453	-0.5046
C	79.3613	-17.4048	-0.3303	O	81.4144	-13.9342	0.5159
C	79.5036	-18.6419	0.4445	H	83.6802	-15.846	-0.6844
C	78.4361	-19.4011	0.6962	H	81.6319	-16.5265	1.273
C	77.1114	-19.0002	0.2104	H	80.2016	-15.461	-0.5234
C	76.9757	-17.8751	-0.4869	H	80.8159	-16.6576	-1.6896
C	78.1517	-17.0459	-0.7694	H	80.4838	-18.9416	0.8154
C	93.8161	-19.8435	-0.4309	H	78.5487	-20.3237	1.2657
C	94.8027	-18.8545	-0.0037	H	76.241	-19.622	0.4199
C	94.6444	-17.4706	-0.4461	H	75.993	-17.5706	-0.8471
C	95.55	-16.549	-0.1341	H	78.0377	-16.1267	-1.344
C	96.7205	-16.9195	0.6589	H	93.7734	-17.1909	-1.0388
C	96.8677	-18.176	1.0723	H	95.42	-15.5198	-0.4688
C	95.8653	-19.1911	0.7249	H	97.4702	-16.1694	0.9108
O	93.0018	-19.4033	-1.2401	H	97.7356	-18.4525	1.671
O	93.8226	-21.2486	0.0215	H	95.9909	-20.2196	1.0632
C	91.3212	-21.7551	-0.8201	H	93.3031	-22.476	-1.6135
C	92.9178	-22.3423	-0.6027	H	95.115	-24.5174	-2.0939
C	93.3792	-23.7913	0.24	H	96.2029	-23.1552	-1.7333
C	95.0415	-23.8543	0.0166	H	94.9385	-26.4861	-0.9576
C	95.7265	-24.0463	-1.3245	H	95.4758	-27.2364	0.5648
O	96.6529	-24.9858	-0.5703	H	93.4491	-26.2591	1.6904
C	95.6755	-25.1109	0.5009	H	89.0114	-25.477	0.1988
C	94.9405	-26.3931	0.1284	H	89.0147	-20.907	-0.6618
C	93.5043	-26.4329	0.6157	H	90.9995	-20.1142	0.7556
C	92.7022	-25.3353	-0.1784	H	90.9371	-21.7543	1.4451
C	91.1867	-25.5751	0.2834	H	88.6243	-23.1608	-2.925
C	89.8501	-25.1162	-0.3965	H	89.4659	-21.6269	-3.2502
C	89.7716	-23.59	-0.4851	H	88.4271	-21.7849	-1.8138
C	89.1142	-22.8044	0.4592	H	90.4363	-24.1171	-3.3281
C	89.1781	-21.1673	0.384	H	91.9962	-23.9355	-2.491
C	90.659	-21.1365	0.5913	H	91.3761	-22.6174	-3.5136
C	90.3812	-22.8151	-1.6164	H	87.9738	-22.6274	2.2646
O	91.0401	-26.4207	1.1737	H	89.1459	-23.9664	2.2593
C	89.1245	-22.3064	-2.4693	H	87.6396	-24.0986	1.3207
C	91.0985	-23.4139	-2.8232	H	92.368	-25.0654	-2.314
C	88.4197	-23.4171	1.6601	H	92.4827	-26.767	-1.8056
C	92.8903	-25.7662	-1.6628	H	93.9521	-25.7681	-1.9093
O	88.2273	-20.3345	1.1685	H	85.6659	-18.9214	-0.6819
C	87.012	-20.3183	0.3045	H	84.6464	-19.8386	2.0579
C	85.8608	-19.2286	0.3457	H	85.5522	-21.919	-0.2968
C	84.4424	-19.6199	1.0098	H	84.436	-23.9005	-1.0882
O	87.0279	-21.0228	-0.7089	H	81.9045	-24.1065	-0.8865
C	83.7208	-20.8966	0.4245	H	80.6319	-22.318	0.1354
C	84.4699	-22.0003	-0.1962	H	81.8174	-20.2507	1.0309
C	83.8567	-23.0949	-0.6371	H	83.5571	-17.7771	1.8767
C	82.4126	-23.2158	-0.5169	H	86.6238	-18.364	2.0223
C	81.715	-22.2334	0.0466	H	90.595	-25.3783	-2.2665
C	82.3978	-21.0373	0.5488	H	93.0199	-27.8526	-0.7683
N	83.5239	-18.3872	0.9983	H	92.0106	-19.9731	-1.4438
O	86.354	-18.0868	1.0461	H	98.0428	-23.7255	-0.3217
O	89.7755	-25.6964	-1.6922	H	98.9845	-23.271	1.1183
O	92.9786	-27.7227	0.2728	H	98.8602	-22.1461	-0.2553
O	91.3787	-20.7407	-1.7815	H	83.0427	-12.254	-0.2816
O	95.62	-22.6622	0.7723	H	83.5834	-13.344	-1.5802
C	97.0773	-22.2767	0.952	H	84.3912	-13.3793	0.0052
O	97.2992	-21.3287	1.7082				

3. Supplementary figures

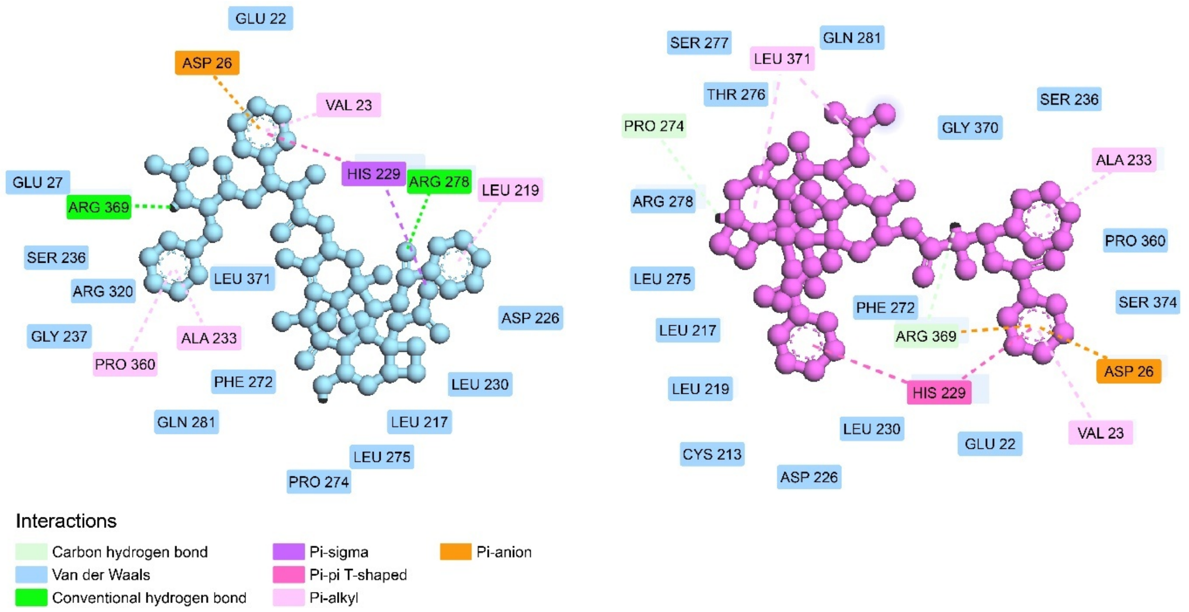

Figure S1. Visualizing interaction residue with Discovery Studio 2022 software on PTX (left) and PTXm (right).

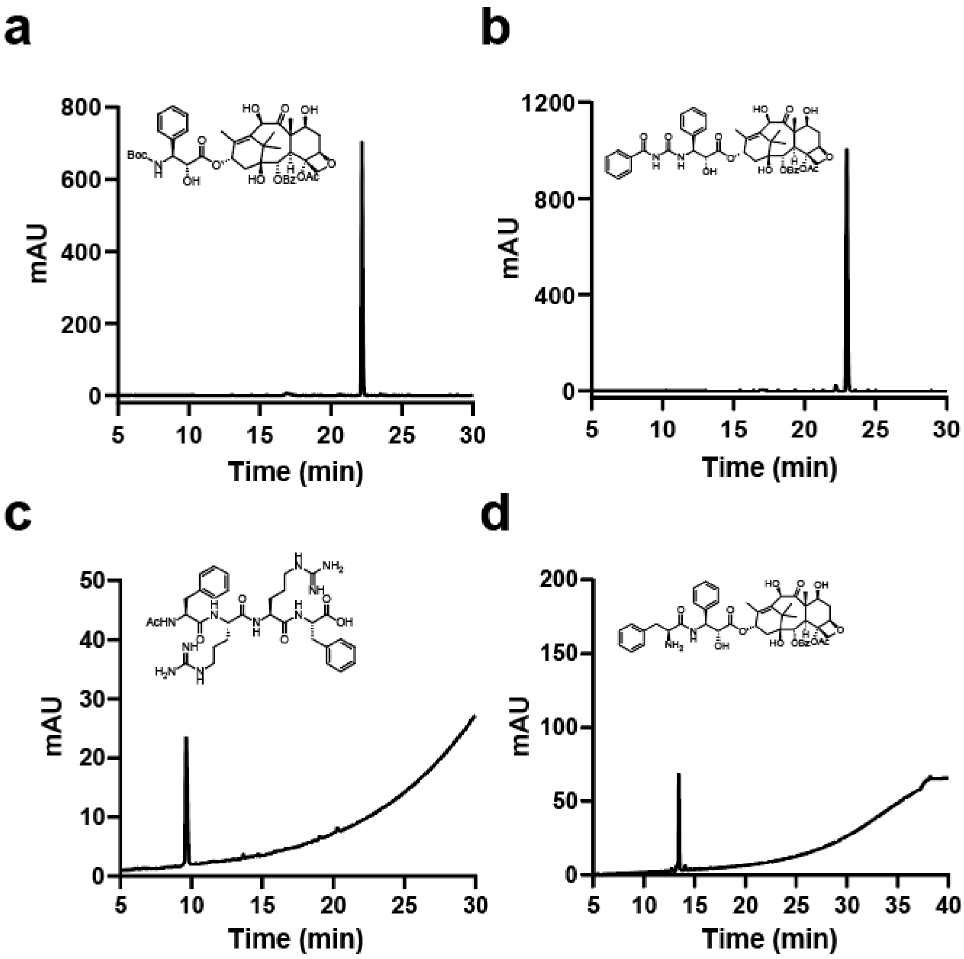

Figure S2. High-performance liquid chromatography (HPLC) data of DTX (**a**), PTX (**b**), (Ac)FRRF (**c**) and PTXm (**d**) were analyzed for verifying synthesis and purification, as well as for comparative purposes.

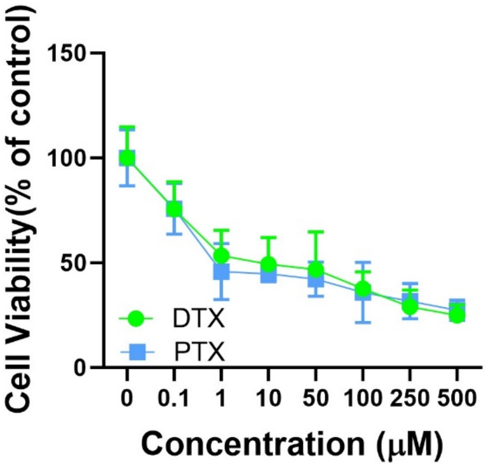

Figure S3. The enhanced solubility of DTX and PTX in 3% DMSO solvent resulted in observed cytotoxic effects within the concentration range of 0.1–500 μM in Hep G2 cancer cells (n = 6).

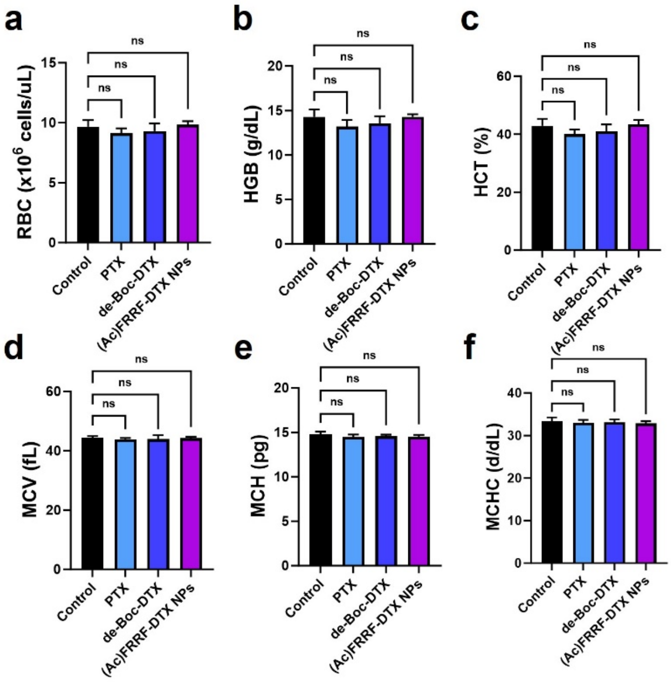

Figure S4. Blood toxicity assessment of (Ac)FRRF-DTX. Mice in each group were administered daily for one week (PTX, de-Boc-DTX: 1.5 mg/kg, (Ac)FRRF-DTX: 3 mg/kg), and peripheral blood analysis was performed for: a) RBC; b) HGB; c) HCT; d) MCV; e) MCH; f) MCHC. The drug toxicity results are presented (n = 5 mice per group).

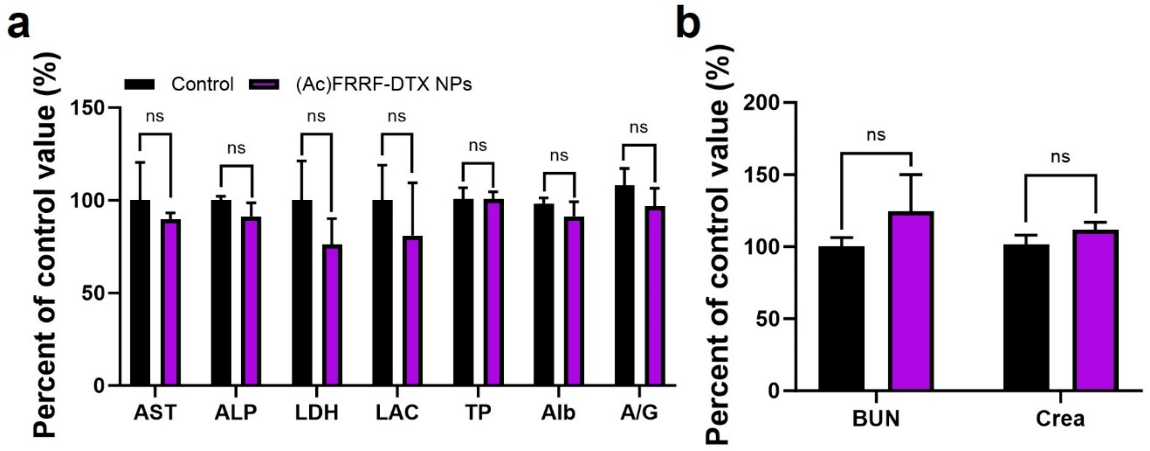

Figure S5. The data comparing the toxicity of (Ac)FRRF-DTX in the liver (a) and kidney (b) with that in the control group (n = 3).

4. Copies of spectra (HRMS, ^{1}H NMR, and ^{13}C NMR)

High resolution mass spectrometry (HRMS) data of **3**, **5**, **6**, and **S1**

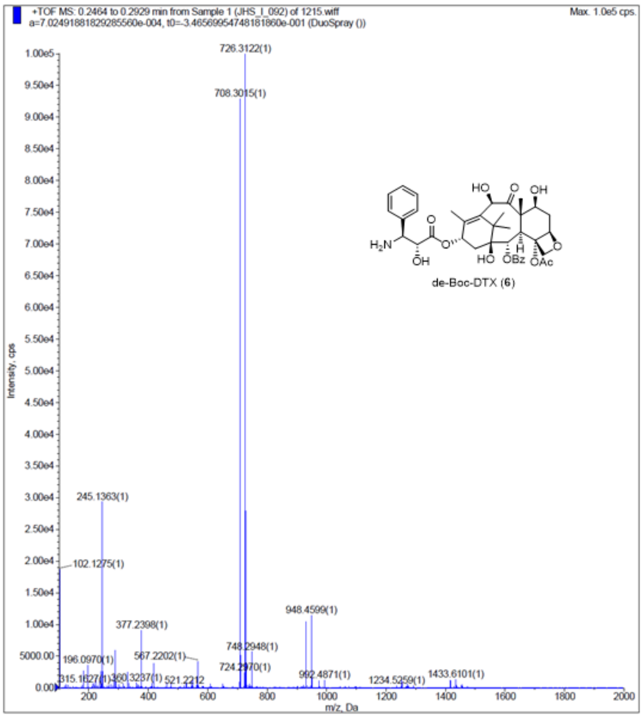

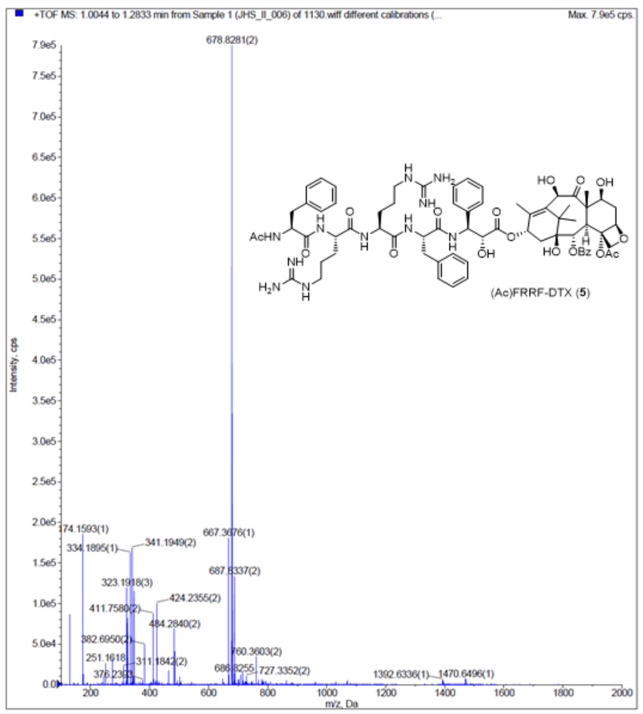

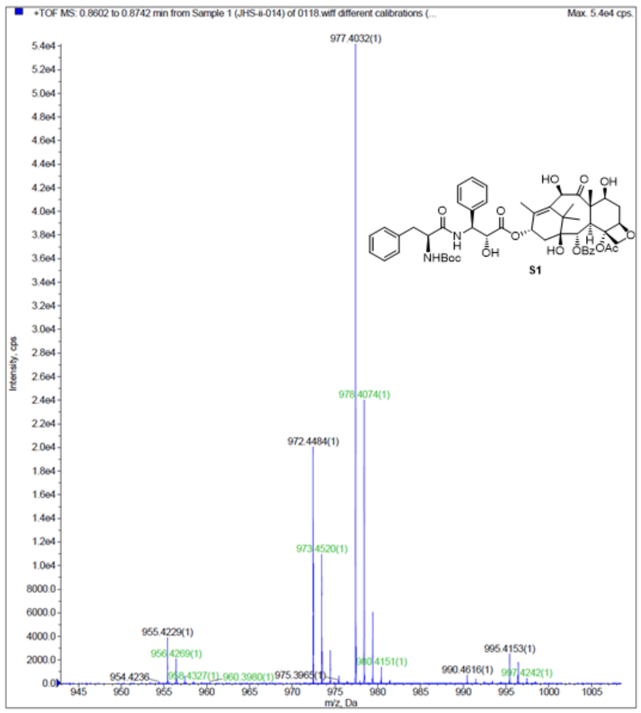

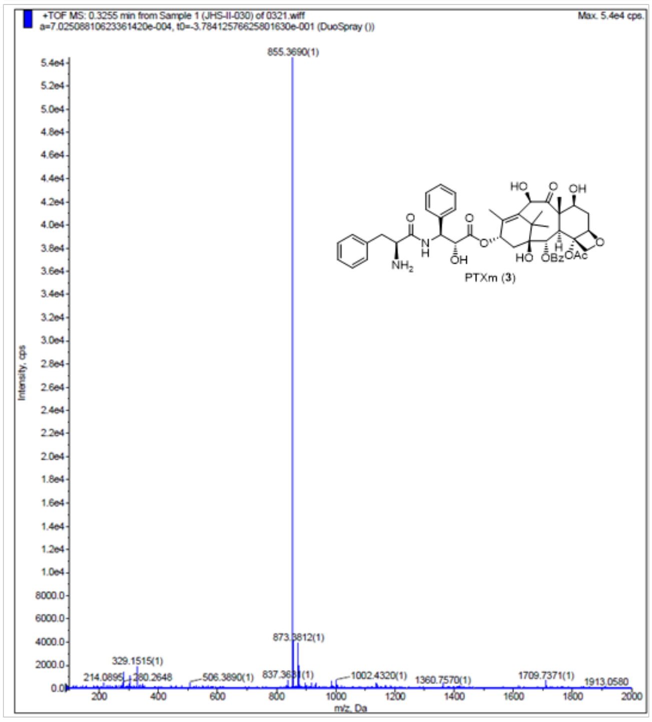

^{1}H NMR (400 MHz, CD_3OD) of **6**

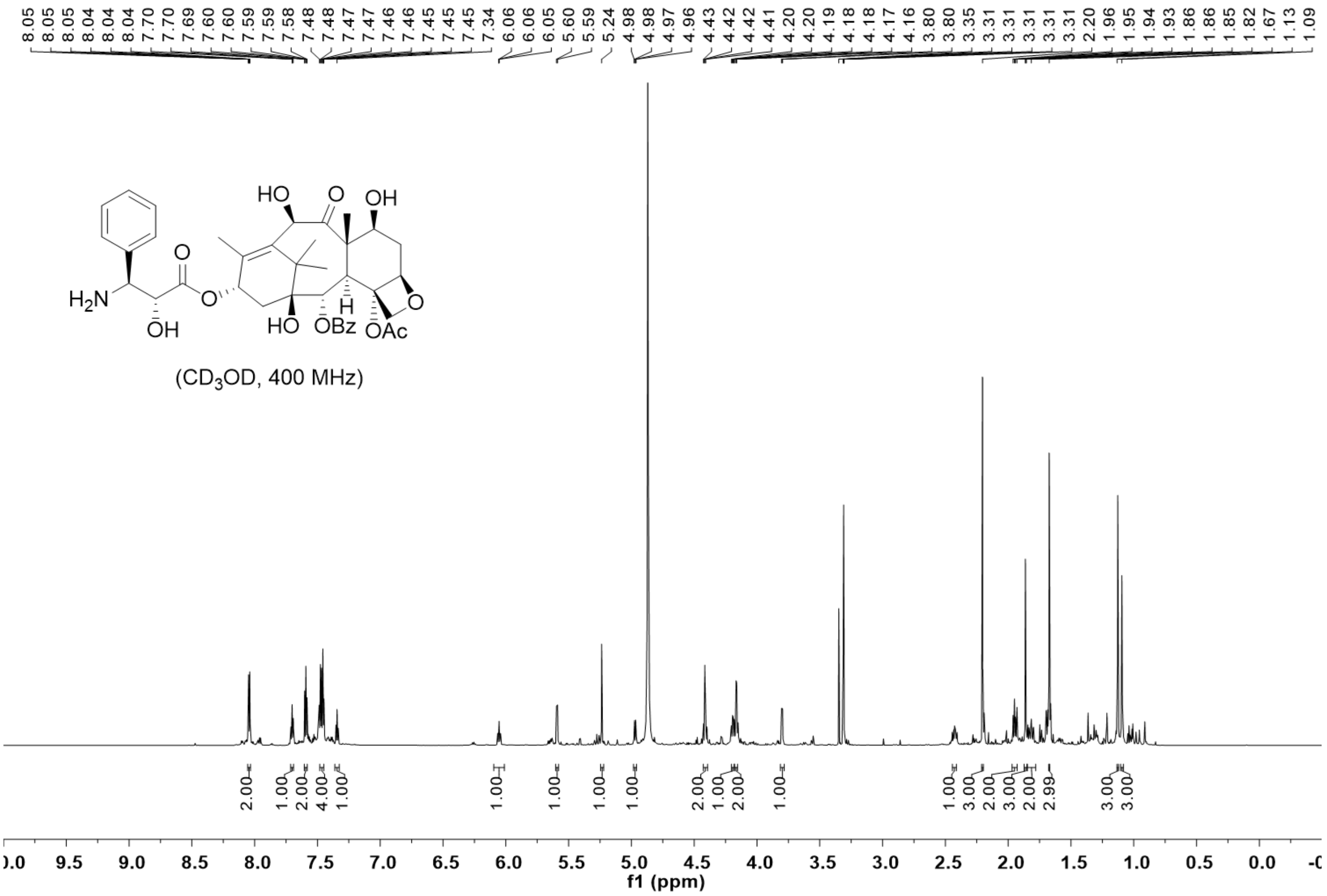

^{13}C NMR (200 MHz, CD_3OD) of **6**

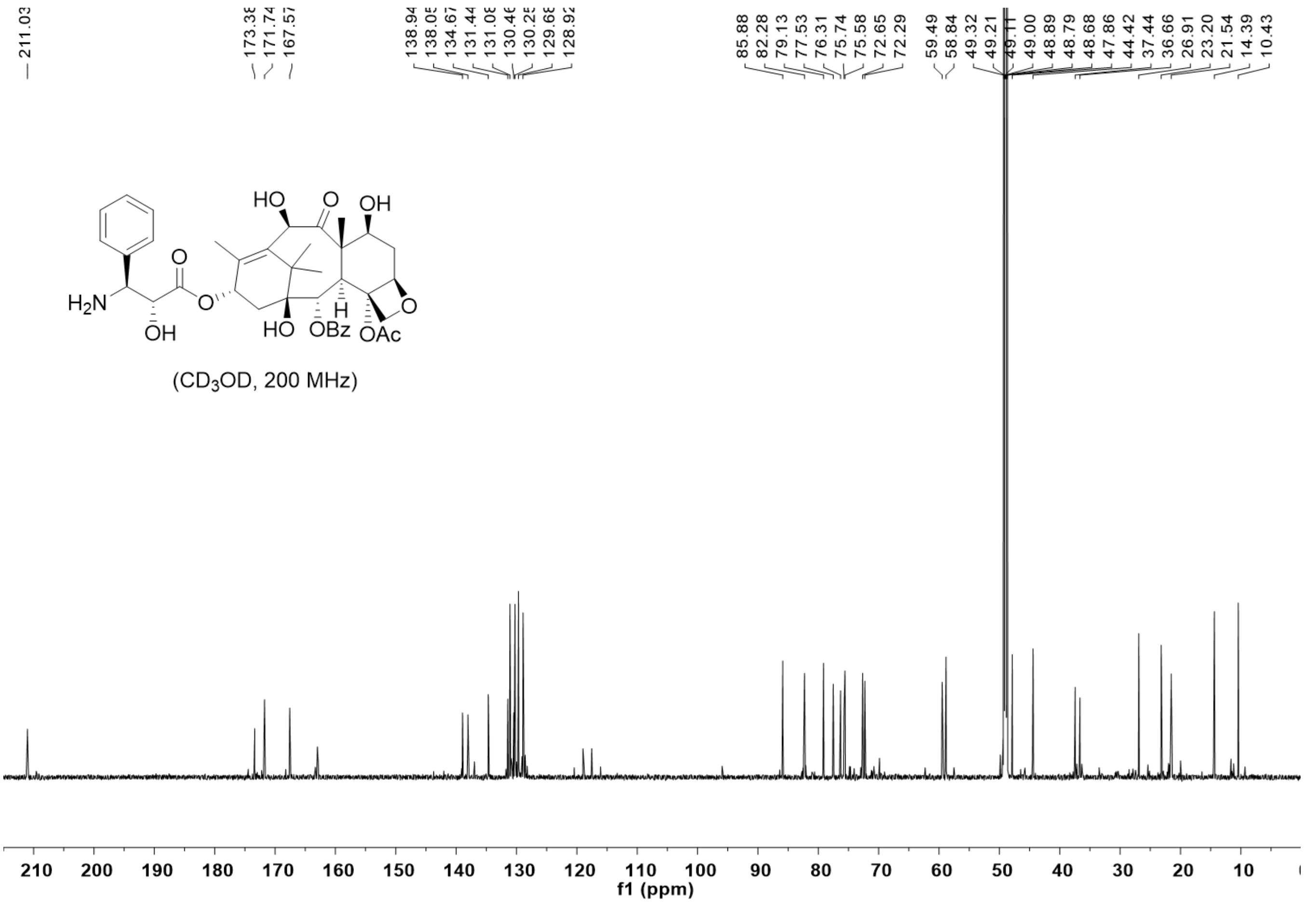

^{1}H NMR (600 MHz, D_2O) of **4**

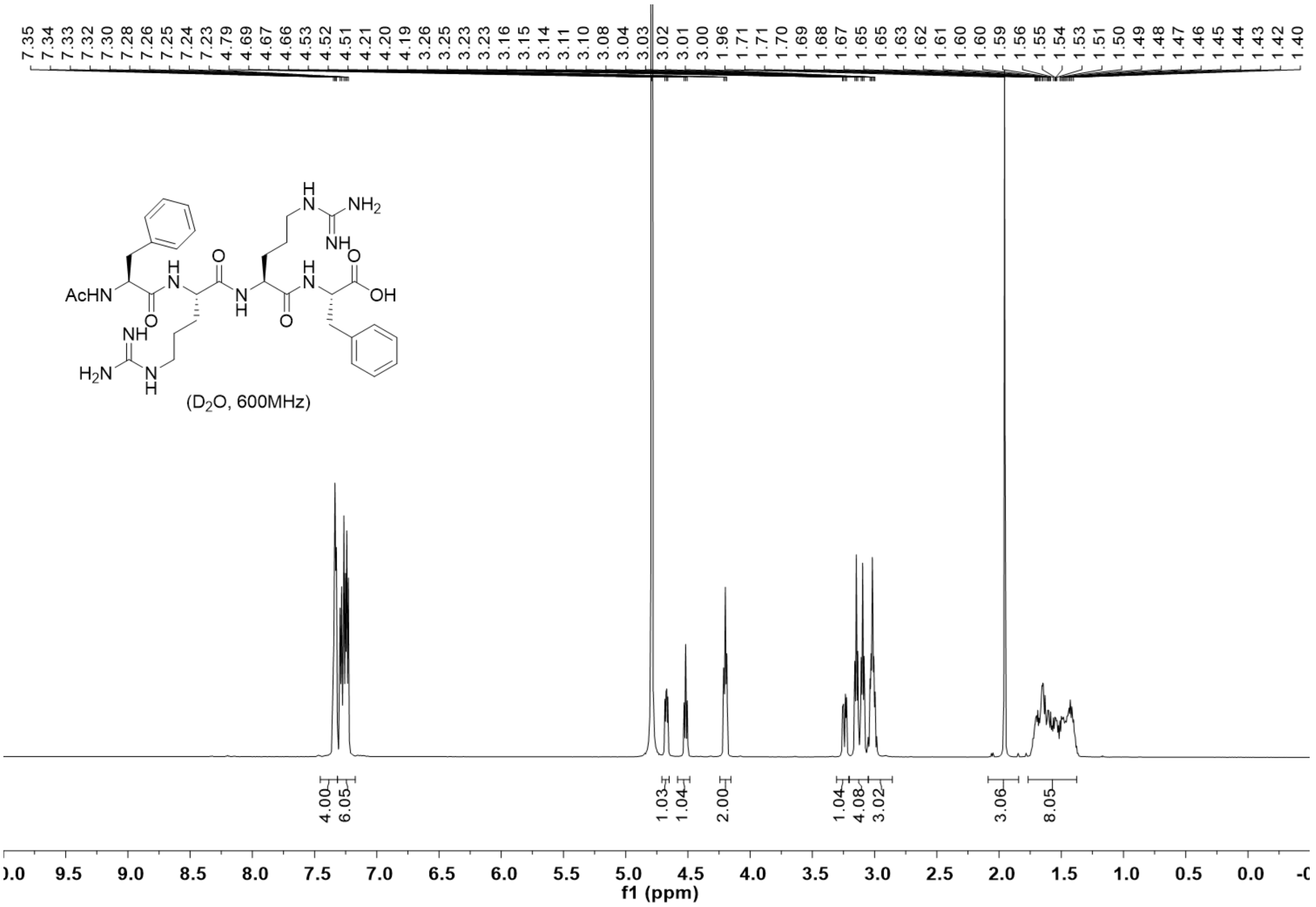

^{13}C NMR (150 MHz, D_2O) of **4**

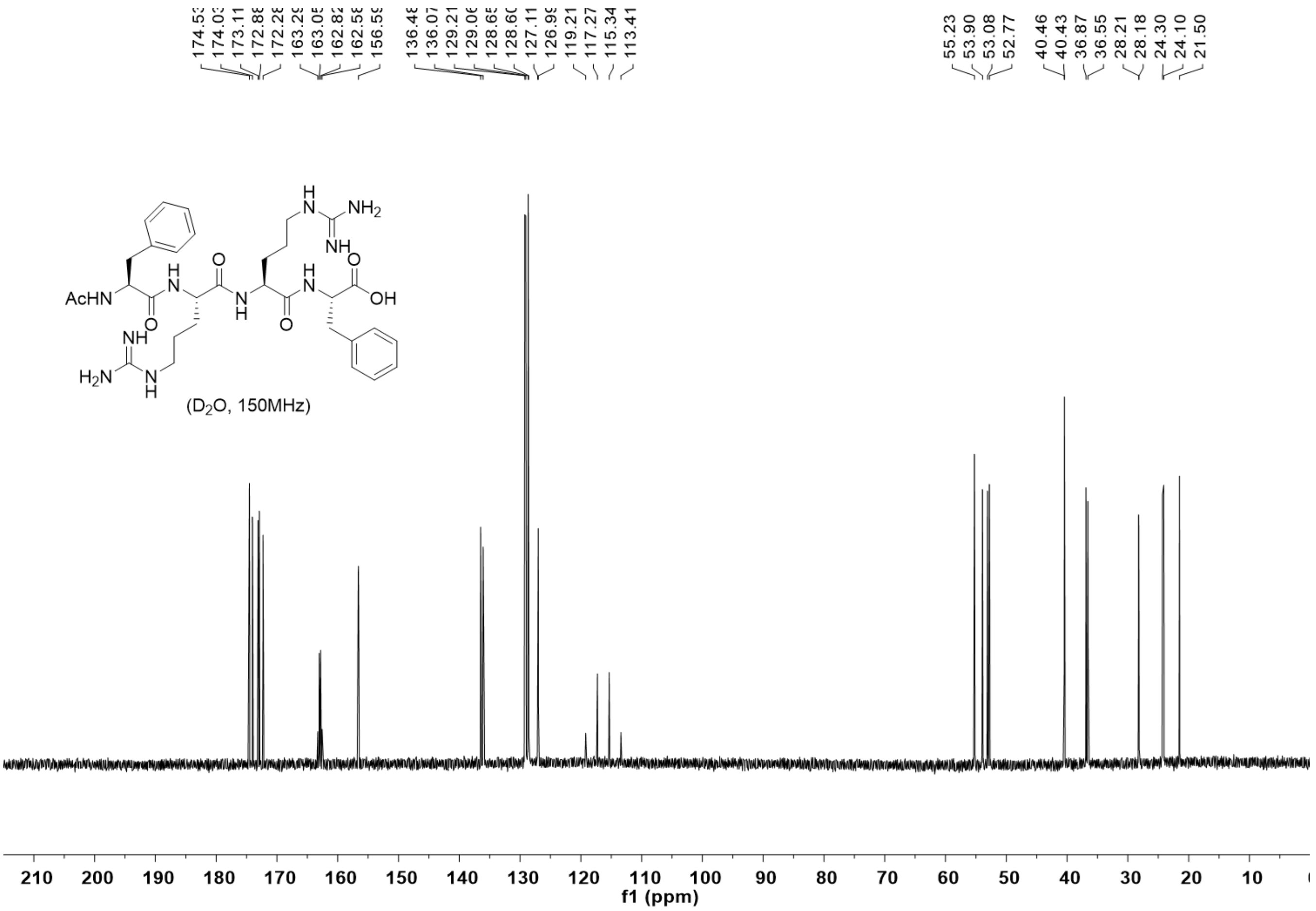

^{1}H NMR (800 MHz, CD_3OD) of **5**

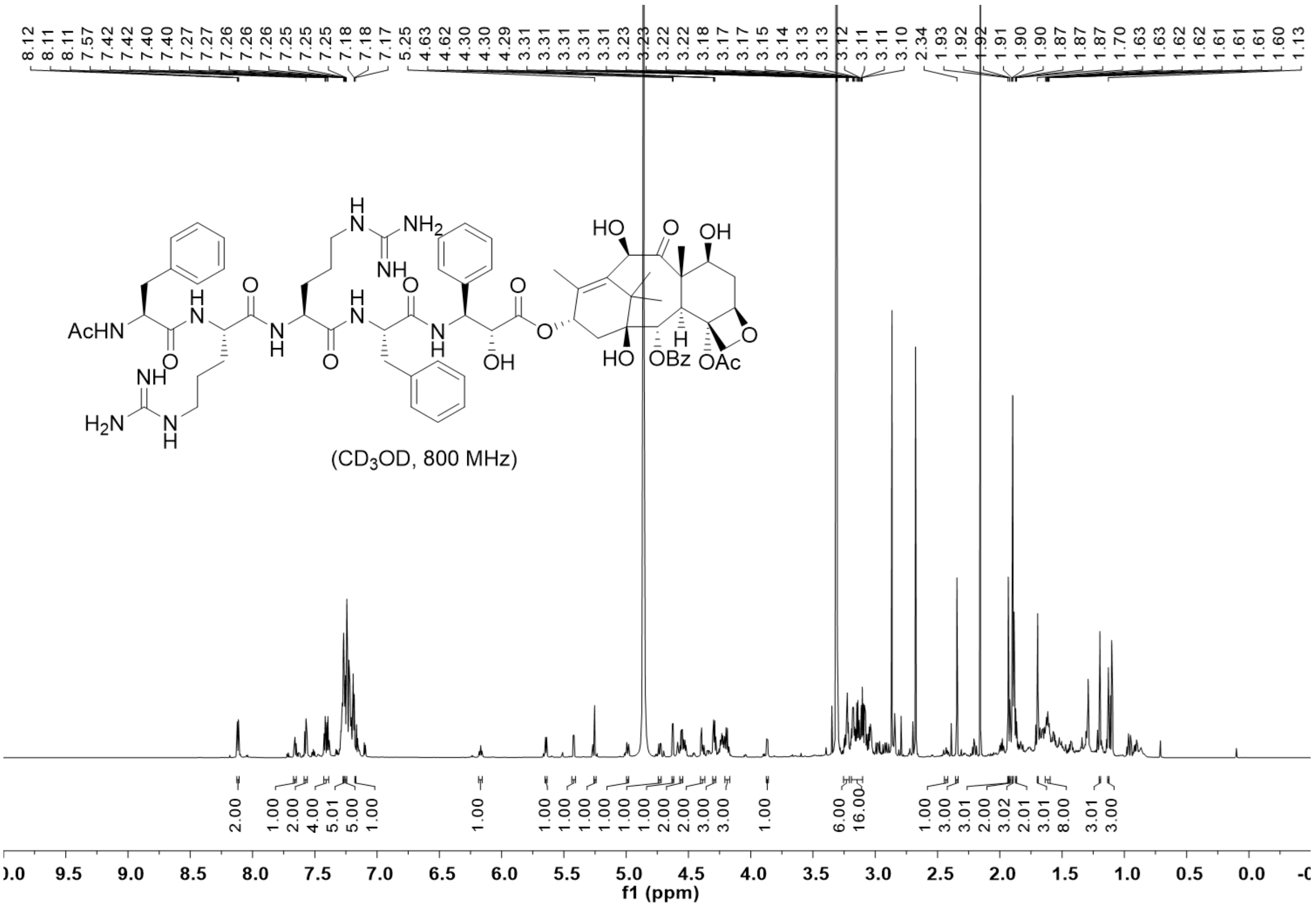

^{13}C NMR (200 MHz, CD_3OD) of **5**

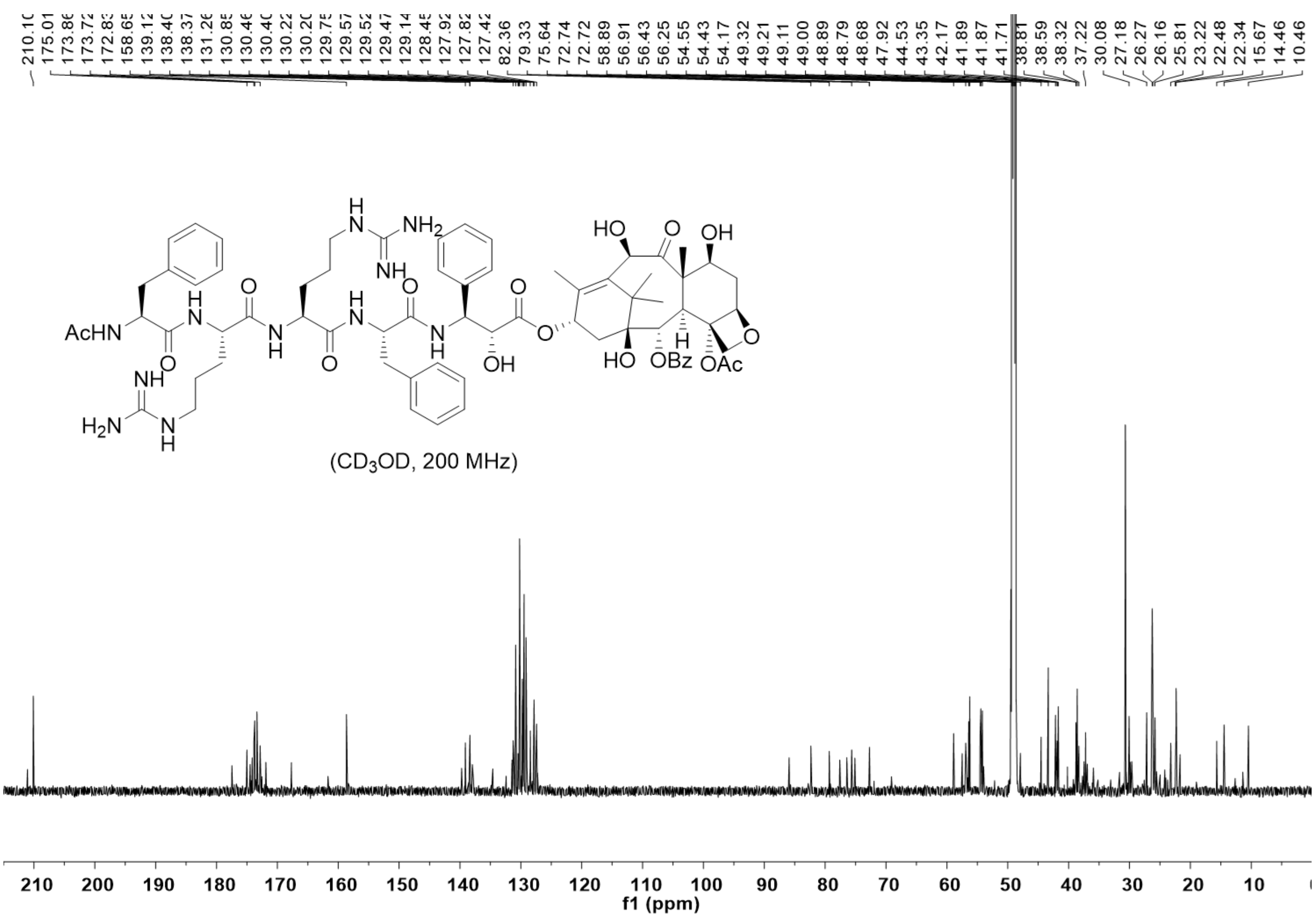

^{1}H NMR (600 MHz, CD_3OD) of **S1**

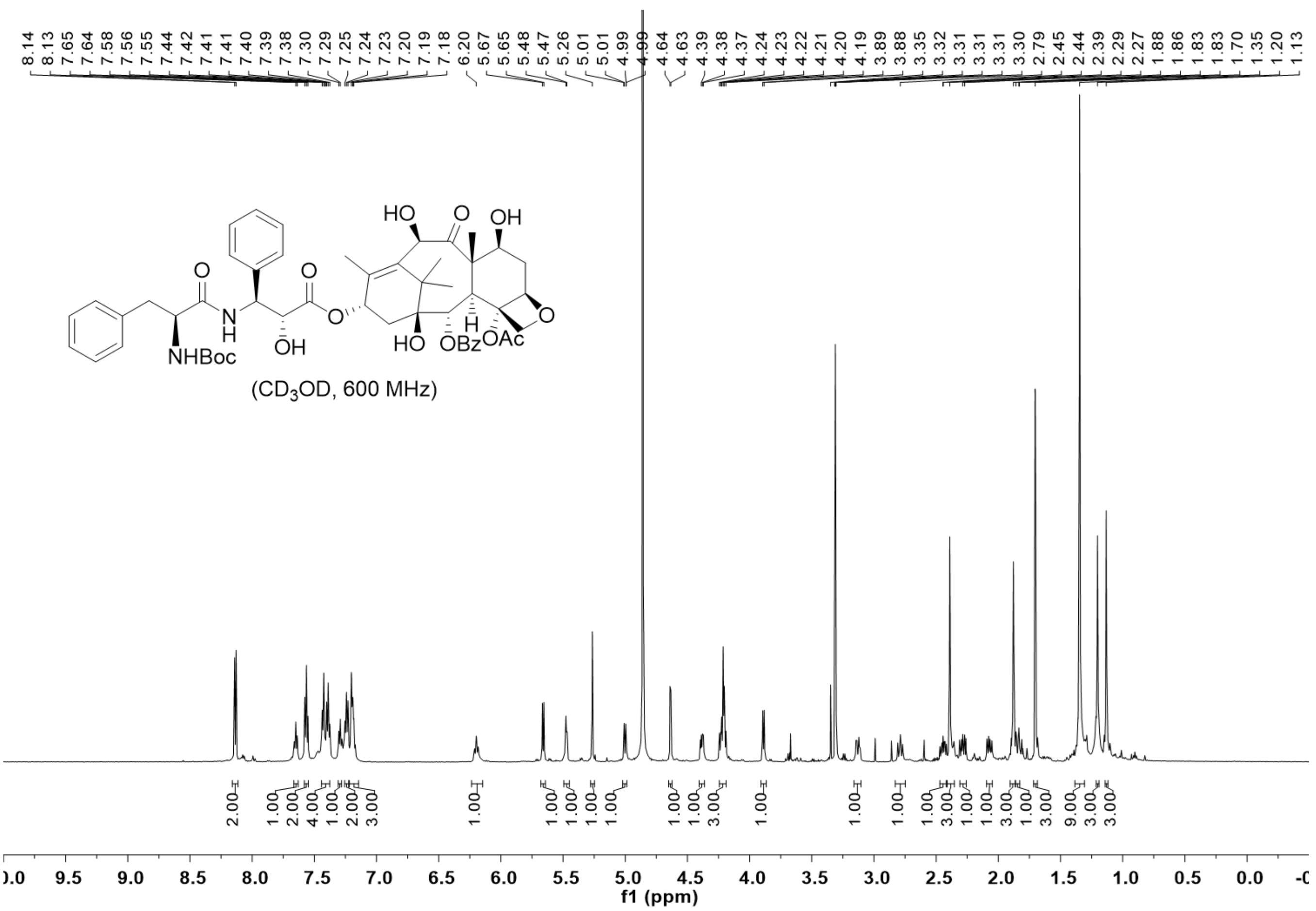

^{13}C NMR (150 MHz, CD_3OD) of **S1**

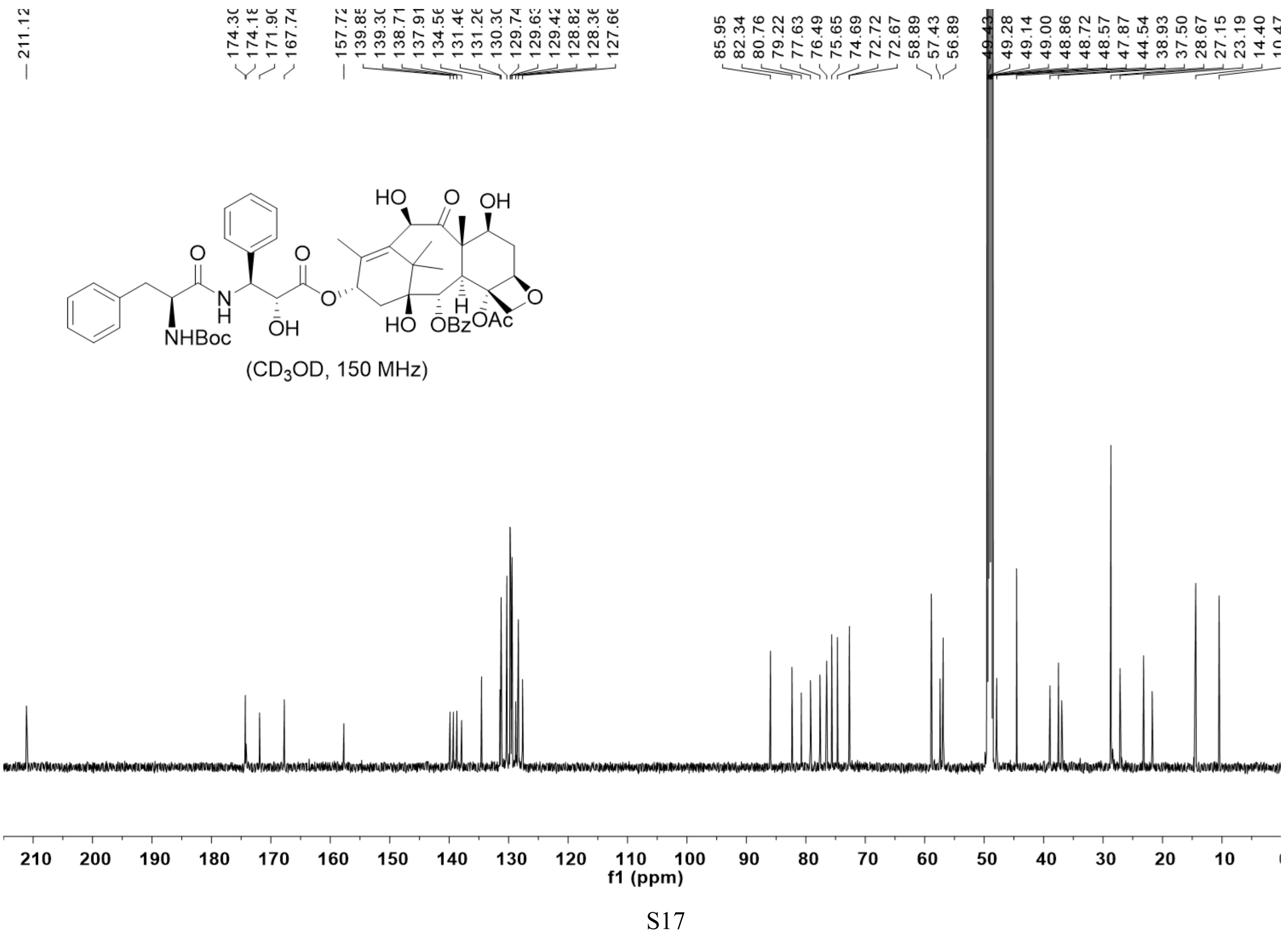

^{1}H NMR (600 MHz, CD_3OD) of **3**

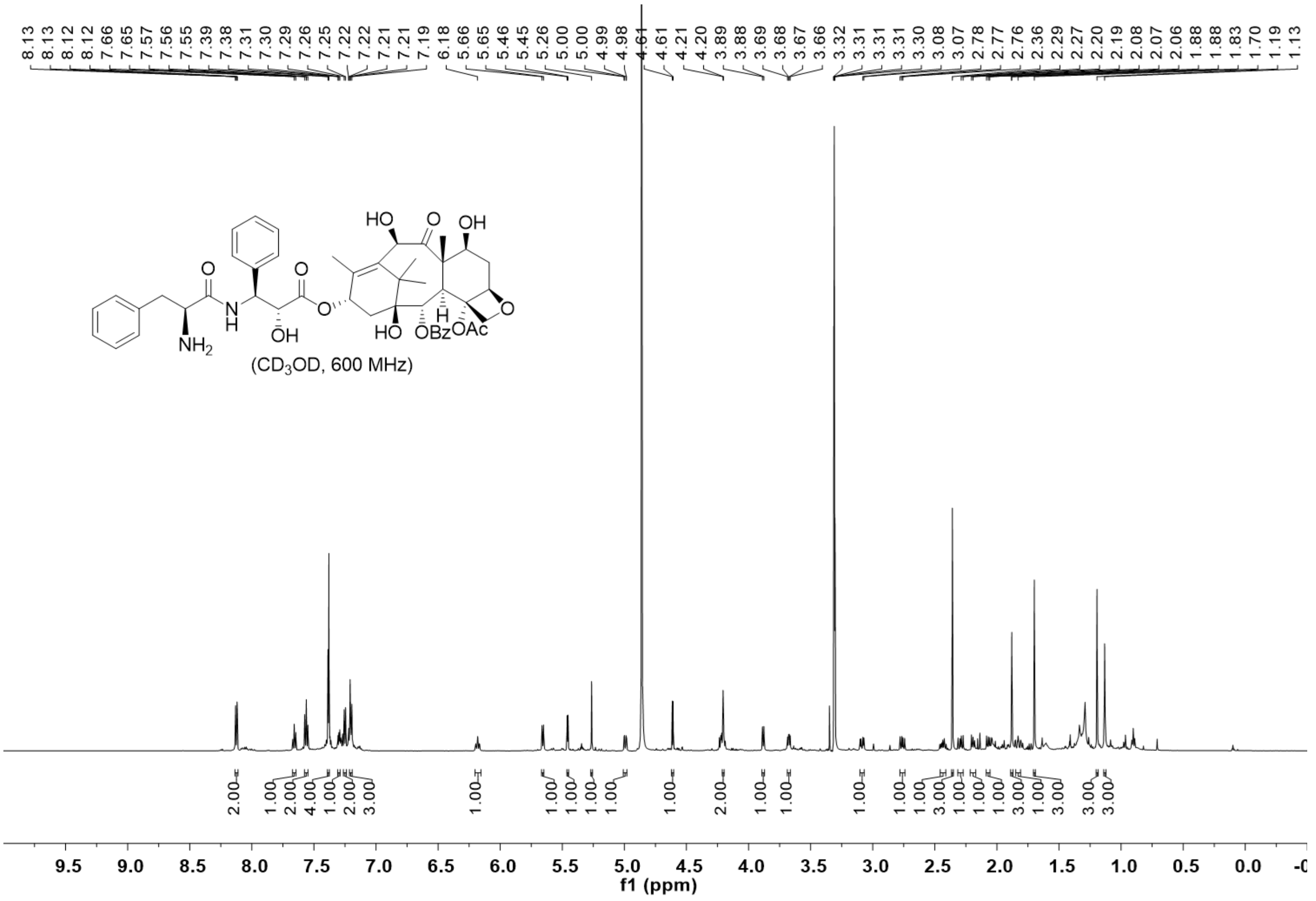

^{13}C NMR (150 MHz, CD_3OD) of **3**

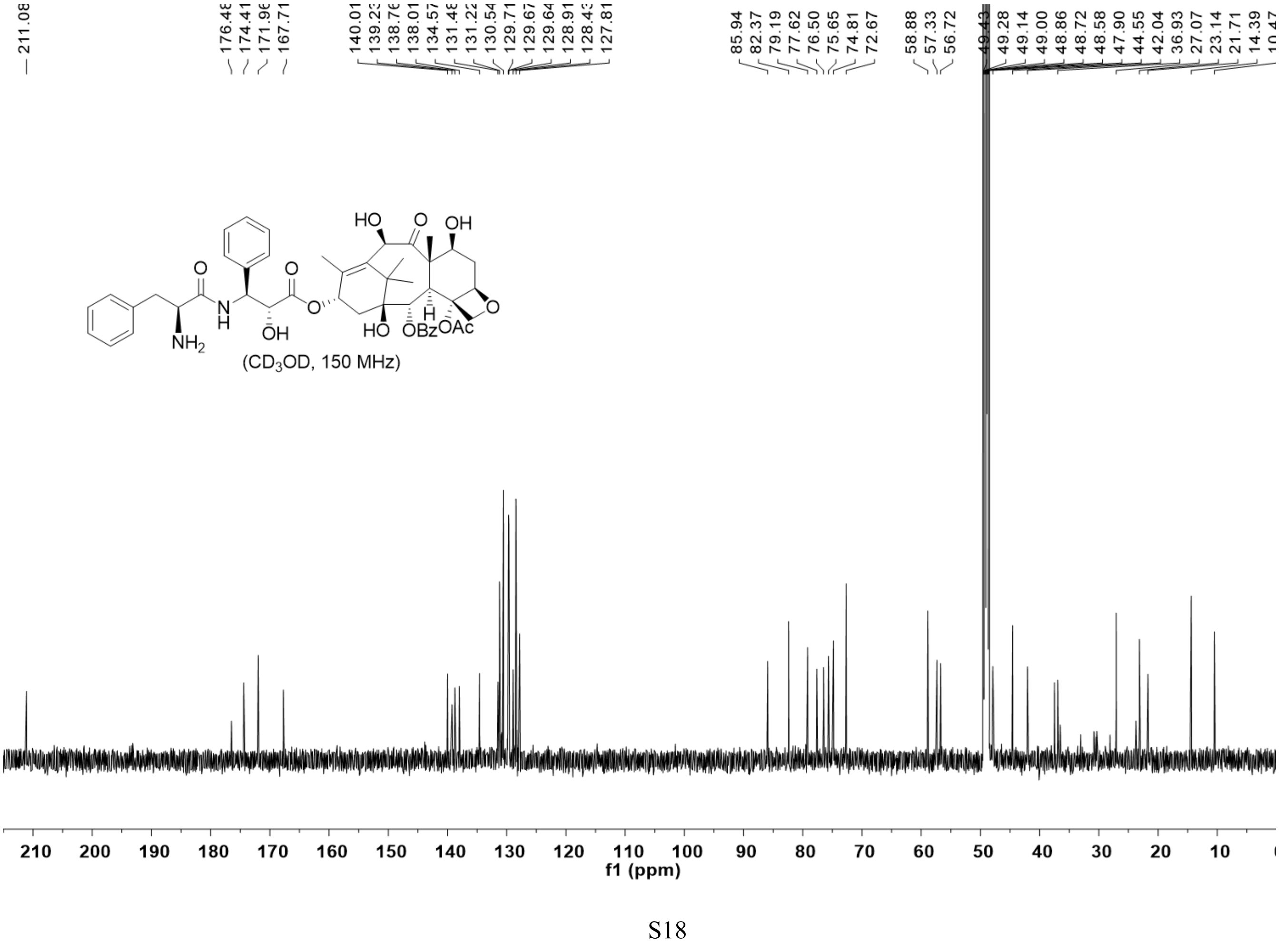

Supplement: Supplementary file 1 — Supplementary Material 1 [file 40580_2025_487_MOESM1_ESM.pdf]
